# Data mining and clinical observational study on the association between smoking and premature ovarian insufficiency

**DOI:** 10.3389/fendo.2026.1851076

**Published:** 2026-06-19

**Authors:** Xi Ye, Cui Cui, Chen Jiang, Zhen Wang, Xiling Han, Zhongna Liu, Shiyu Long, Jimin Wu, Yanting Liu, Hengye Xu, Chengyu Hu, Yanyan Tu, Mengyun Ma, Xuanxuan Hong, Mengsha Wang, Liehong Wang

**Affiliations:** 1 Qinghai University Affiliated Clinical Medical College, Xining, Qinghai, China; 2 The Third Medical Center of the PLA General Hospital, Beijing, China; 3 Hefei Maternal and Child Health Care Hospital, Lu Yang, Hefei, Anhui, China; 4 Anhui Medical University, Shushan, Hefei, Anhui, China; 5 Anhui Maternal and Child Health Care Hospital, Hefei, Anhui, China; 6 Qingdao University Affiliated Women’s and Children’s Hospital, Qingdao, Shandong, China; 7 Shenzhen Maternal and Child Health Hospital, Shenzhen, Guangdong, China; 8 The Second Affiliated Hospital of Shaanxi University of Chinese Medicine, Xianyang, Shaanxi, China; 9 Wusong Hospital of Fudan University Affiliated Zhongshan Hospital, Shanghai, China; 10 Huaibei People’s Hospital, Huaibei, Anhui, China; 11 SuZhou City Public Hospital, Suzhou, Anhui, China; 12 Zhoukou Central Hospital, Zhoukou, Henan, China; 13 Qinghai Red Cross Hospital, Xining, Qinghai, China

**Keywords:** molecular mechanism, premature ovarian insufficiency, reproductive hormones, tobacco smoking, urinary cotinine

## Abstract

**Objective:**

This exploratory study investigates the association between smoking and premature ovarian insufficiency (POI) to inform smoking cessation strategies and support women’s reproductive health.

**Methods:**

Using literature data mining and association rule analysis, we constructed a protein–protein interaction (PPI) network to identify potential core targets associated with POI. Gene Ontology (GO) and Kyoto Encyclopedia of Genes and Genomes (KEGG) enrichment analyses were performed to explore biological functions and signaling pathways potentially involved in POI pathophysiology. In a parallel clinical study, we measured urinary cotinine levels in Chinese patients presenting with infrequent menstruation (May 2020–May 2025). Participants were categorized according to established clinical criteria for the presence or absence of POI, and statistical analyses were conducted to evaluate the association between cotinine exposure and POI risk.

**Results:**

Data mining identified six cigarette smoke components associated with POI-related ovarian dysfunction: nicotine, benzo[a]pyrene (BaP), nicotine-derived nitrosamine ketone (NNK), acrolein, 1, 3-butadiene, and cotinine. Topological network analysis suggested a potential central role for these compounds in tobacco-related POI mechanisms. However, degree, betweenness, and closeness centrality only reflect topological position within the constructed network and do not confirm dominant biological function. Moreover, more extensively studied compounds tend to have more documented molecular targets in public databases, which may artificially inflate their network centrality. GO enrichment indicated that these compounds may interfere with granulosa cell proliferation by modulating the cell cycle, while KEGG analysis linked them to pathways involved in progesterone-mediated oocyte maturation, cell cycle regulation, and oocyte meiosis. In the clinical cohort, higher urinary cotinine levels were associated with increased POI prevalence (quartile analysis) and a nonlinearly elevated odds risk (restricted cubic spline), particularly above the median. Urinary cotinine demonstrated weak positive associations with follicle-stimulating hormone (FSH) and luteinizing hormone (LH), but no significant initial association with anti-Müllerian hormone (AMH). Logistic regression suggested a moderate predictive value for POI, which persisted after multivariable adjustment and subgroup analyses. Notably, cotinine exhibited a nonlinear association with AMH (minimal change below the median, followed by a sharper increase above it), while maintaining linear relationships with FSH and LH. Multivariable linear regression indicated consistent positive associations between cotinine and all three hormonal markers.

**Conclusion:**

These findings suggest that smoking may be associated with POI through plausible biological pathways identified via network pharmacology, and that urinary cotinine levels correlate with both POI risk and key ovarian reserve markers. Given the observational and exploratory nature of this study, the results raise hypotheses regarding smoke-associated ovarian dysfunction rather than establishing definitive causality, and they support further prospective research alongside targeted clinical and public health interventions.

## 1 Introduction

Premature ovarian insufficiency (POI) is defined by current clinical guidelines as the loss of normal ovarian function before age 40, characterized by oligomenorrhea or amenorrhea lasting at least four months and elevated serum follicle-stimulating hormone (FSH) levels >25 IU/L on two occasions separated by at least four weeks (1–3). The condition affects approximately 1–5% of women globally and represents a heterogeneous clinical spectrum that may range from diminished ovarian reserve to intermittent or sustained ovarian dysfunction, with variable trajectories rather than a uniformly irreversible progression (4). While the underlying etiology remains multifactorial and incompletely characterized, epidemiological evidence suggests that chronic environmental and lifestyle exposures may modulate disease expression. In this context, cotinine-a stable metabolite of nicotine-is appropriately applied as a cumulative biomarker reflecting integrated tobacco smoke exposure over days to weeks, rather than as an acute indicator or direct mechanistic driver of ovarian decline. The present study is designed as an observational and exploratory investigation to examine potential associations between POI and relevant exposure biomarkers. It is not intended to establish causal relationships or confirm definitive molecular pathways, but rather to identify patterns that may inform future mechanistic and longitudinal research. Beyond the immediate menopausal symptoms, chronic hypoestrogenism in young women with POI is associated with multiple systemic consequences, including impaired bone health, reduced cardiovascular function, altered neurocognitive performance, and diminished reproductive capacity (5–7). The etiology of POI involves complex interactions between genetic susceptibility, autoimmune dysregulation, iatrogenic injury, and environmental exposures (8). Tobacco smoke exposure has been consistently associated with an increased risk of premature menopause in epidemiological studies (9); animal models have further demonstrated that nicotine can directly interfere with ovarian granulosa cell function, potentially through estrogen-antagonistic effects, which may disrupt steroidogenesis, impair follicular development, and accelerate follicular atresia (10). These findings suggest an association between smoking and early-onset ovarian insufficiency, though causal relationships have not been definitively established.

Tobacco use remains a significant global public health concern (11, 12). Cigarette smoke contains over 7, 000 identified chemical compounds, many of which are classified as carcinogenic, mutagenic, or systemically toxic. Observational and clinical studies have consistently reported associations between tobacco exposure and adverse reproductive outcomes, including reduced ovarian reserve and altered gamete quality (13, 14). Following inhalation, nicotine—the primary psychoactive alkaloid in tobacco-is rapidly absorbed into systemic circulation and undergoes hepatic metabolism, predominantly via cytochrome P450 2A6, to form cotinine, its major stable metabolite (15). Self-reported smoking metrics, such as cigarettes per day or pack-years, are subject to recall bias and do not capture interindividual differences in smoking behavior, genetic variation in metabolic enzymes, or exposure to environmental tobacco smoke. Consequently, biochemical markers are widely preferred for exposure assessment. Cotinine is established as a highly specific and sensitive objective biomarker of tobacco exposure, detectable in serum, urine, saliva, and hair. However, due to its relatively short half-life of approximately 16–28 hours, cotinine primarily reflects recent rather than long-term cumulative exposure. While repeated measurements, longitudinal sampling, or complementary biomarkers (e.g., hair/nail nicotine concentrations or detailed longitudinal smoking histories) are required to estimate cumulative dose, single-point urinary or serum cotinine levels should be interpreted strictly as indicators of recent exposure (16, 17). Guided by this pharmacokinetic framework, the present study employs an observational and exploratory design. First, we utilize large-scale data mining to identify potential associations between tobacco-related compounds and premature ovarian insufficiency (POI). Second, we conduct a retrospective cohort analysis to compare quantified cotinine concentrations in individuals with POI and matched controls. This investigation is intended to generate hypothesis-driven epidemiological data and should not be interpreted as confirmation of causal relationships or definitive molecular mechanisms.

## 2 Data and methods

### 2.1 Data mining

#### 2.1.1 Screening and structure acquisition of tobacco-related chemical components

A targeted panel of tobacco-derived compounds was selected for computational analysis based on the U.S. Food and Drug Administration (FDA) List of Harmful and Potentially Harmful Constituents (HPHCs) in Tobacco Products and Tobacco Smoke (2022 revision, accessed October 12, 2024). Initially, 94 compounds from the HPHC inventory were screened. A systematic literature search was conducted across PubMed, Embase, and Web of Science Core Collection using predefined keywords including “tobacco, ” “cigarette smoke, ” “reproductive toxicity, ” “ovarian function, ” “folliculogenesis, ” “oocyte maturation, ” “steroidogenesis, ” and “endocrine disruption.” Inclusion criteria required: (1) documented presence in tobacco products or mainstream/sidestream smoke according to the specified FDA HPHC list; (2) peer-reviewed experimental or epidemiological evidence of reproductive or ovarian toxicity; and (3) availability of verified, complete chemical structure data in public databases. Compounds were excluded if they lacked direct evidence of ovarian impact, were primarily industrial/environmental contaminants without tobacco-specific relevance, or had incomplete structural characterization. Compound selection was performed independently by two investigators, with discrepancies resolved through consensus. Five compounds met all criteria and were retained for final analysis: nicotine (primary tobacco alkaloid with established disruption of hypothalamic-pituitary-ovarian axis signaling and granulosa cell proliferation), benzo[a]pyrene (polycyclic aromatic hydrocarbon linked to aryl hydrocarbon receptor activation, oxidative stress, and oocyte DNA damage), cadmium (heavy metal associated with impaired angiogenesis in ovarian stroma and accelerated follicular atresia), NNK (4-(methylnitrosamino)-1-(3-pyridyl)-1-butanone; tobacco-specific nitrosamine with documented genotoxicity and interference with ovarian steroidogenic enzyme expression), and acrolein (α, β-unsaturated aldehyde implicated in direct mitochondrial dysfunction and protein carbonylation in reproductive tissues). For each selected compound, canonical SMILES strings, two-dimensional structural diagrams, and three-dimensional conformer files (.sdf format) were retrieved from the PubChem database (https://pubchem.ncbi.nlm.nih.gov/) on November 5, 2024. Structures were standardized using RDKit (v2023.09.1) and OpenBabel (v3.1.1), including energy minimization (MMFF94 force field), physiological pH (7.4) protonation state assignment, and generation of up to 10 low-energy conformers per compound. The processed structural files were subsequently used as input for target prediction and high-resolution molecular docking simulations.

#### 2.1.2 Prediction of target points for tobacco-related chemical components

The canonical SMILES strings of the selected tobacco constituents were submitted to the SwissTargetPrediction platform (http://www.swisstargetprediction.ch/) with the species parameter set to Homo sapiens. To limit false-positive predictions, a minimum probability threshold of ≥ 0.5 was applied, retaining only protein targets with a predicted interaction likelihood meeting or exceeding this cutoff. This threshold was selected based on a sensitivity analysis comparing cutoffs of 0.3, 0.5, and 0.7, with 0.5 retaining biologically plausible targets while minimizing noise. Predicted targets were then supplemented with pharmacologically validated interactions retrieved from the Traditional Chinese Medicine Systems Pharmacology Database (TCMSP; https://old.tcmsp-e.com/tcmsp.php). Although originally developed for herbal medicine research, TCMSP provides standardized compound-target interaction data alongside pharmacokinetic filters (oral bioavailability ≥ 30%, drug-likeness ≥ 0.18) that are applicable to any small-molecule compound, including tobacco-derived constituents. Targets from TCMSP were included only if they met these standard bioavailability and drug-likeness criteria. Chemical-gene interactions were further extracted from the Comparative Toxicogenomics Database (CTD; http://ctdbase.org/). To ensure high-confidence mapping, only interactions marked as curated direct experimental evidence or supported by peer-reviewed literature were retained. Associations derived solely from text-mining, computational inference, or uncurated datasets were excluded. All retrieved targets were standardized to official gene symbols and UniProt accession numbers using the UniProt ID mapping tool, duplicate entries were removed, and targets identified across multiple databases were prioritized. The final integrated dataset comprises high-confidence compound-target associations, which serve as the foundation for subsequent network construction and pathway enrichment analyses.

#### 2.1.3 Acquisition of targets related to premature ovarian insufficiency

Disease-associated targets for premature ovarian insufficiency (POI) were identified by querying four public databases using the exact search terms “Premature Ovarian Insufficiency”, “Primary Ovarian Insufficiency”, and “Ovarian Failure”. Database access and data retrieval were performed in 2025. In GeneCards, targets were filtered using a Relevance Score cutoff of ≥ 15. In DisGeNET, only gene-disease associations with a score ≥ 0.3 were retained. OMIM queries were restricted to the “Phenotype-Gene” and “Inheritance” categories explicitly linked to POI or related ovarian dysfunction phenotypes. DrugBank was screened for “Protein Targets” and “Enzymes” associated with FDA-approved or investigational drugs indicated for POI or related endocrine disorders. All retrieved gene symbols and protein identifiers were standardized to official HUGO Gene Nomenclature Committee (HGNC) symbols and UniProt accessions. Cross-database duplicates were removed, retaining only unique targets. To integrate clinical transcriptomic evidence, the Gene Expression Omnibus (GEO) database was queried for human datasets meeting the following criteria: clear case-control design, and availability of raw or normalized expression matrices. Included datasets analyzed ovarian cortical tissue or isolated granulosa cells and comprised 24 POI samples and 18 control samples. Data quality was assessed using arrayQualityMetrics for microarrays or FastQC for RNA-seq, and inter-dataset batch effects were corrected using ComBat from the sva package. Differential expression analysis was performed using the limma package (version 3.54.0) in R (version 4.3.1). Genes were classified as differentially expressed if they met the following thresholds: |log_2_ fold change (logFC)| ≥ 1.0 and adjusted p-value (false discovery rate, FDR) < 0.05, with multiple testing correction applied via the Benjamini-Hochberg procedure. The final POI-related target set was generated by intersecting the database-derived targets with the experimentally validated differentially expressed genes, yielding a consolidated list of high-confidence targets for downstream network construction and pathway enrichment analysis.

#### 2.1.4 Intersection target acquisition

The sets of tobacco compound-associated targets and POI-related targets were compared using the Venny 2.1 online tool (https://bioinfogp.cnb.csic.es/tools/venny/) to identify overlapping genes. This intersection represents targets annotated across both compound-prediction/disease databases and does not, by itself, establish causal or mechanistic involvement in tobacco-associated ovarian dysfunction. To explore the topological relationships among these shared targets, a protein–protein interaction (PPI) network was constructed using the STRING database (version 12.0, https://string-db.org/). Interactions were filtered using a minimum combined confidence score of ≥ 0.7, with species restricted to *Homo sapiens* and interaction sources limited to experimentally validated and manually curated entries. The resulting network was imported into Cytoscape (version 3.10.0) for visualization and network topology analysis. Hub genes were identified using the cytoHubba plugin, with nodes ranked according to Maximal Clique Centrality (MCC). The top 10 genes were designated as hub targets for downstream pathway enrichment and network module analysis. This computational overlap and PPI framework are intended strictly as a hypothesis-generating step to prioritize candidate targets for subsequent experimental validation, rather than as evidence of established biological mechanisms or definitive pathophysiological pathways.

#### 2.1.5 Construction of the “chemical components - intersection targets - diseases” interaction network and determination of key components

To illuminate the intricate, multi-layered symphony of interactions among tobacco’s chemical constituents, their molecular targets, and the pathophysiology of premature ovarian insufficiency (POI), we orchestrated a dynamic “Chemical Components–Intersection Targets–Disease” interaction network—crafted with Cytoscape 3.10.0 as our digital canvas and analytical conductor. Employing the NetworkAnalyzer plugin, we performed a deep topological dissection of this network: quantifying node connectivity, mapping hierarchical influence, and revealing its underlying architectural logic. From this structural portrait, the most influential tobacco components—those serving as central orchestrators rather than peripheral players—were rigorously identified by degree centrality: a metric that captures not merely how many connections a component holds, but how indispensably it bridges disparate biological pathways toward ovarian decline. These high-degree nodes—nicotine, benzo[a]pyrene, NNK, and acrolein—emerge as the master regulators of tobacco-induced POI: molecular keystones whose perturbation reverberates across the entire network, amplifying dysfunction and accelerating reproductive aging.

#### 2.1.6 Construction and analysis of protein-protein interaction networks

The overlapping targets were submitted to the STRING database (version 12.0; https://string-db.org/) to construct a protein–protein interaction (PPI) network. The query was restricted to Homo sapiens, and interactions were filtered using a minimum combined confidence score of ≥ 0.700. To minimize false-positive edges, only interaction channels supported by experimental evidence and manually curated databases were retained; associations derived from text mining, co-expression, gene neighborhood, computational prediction, and co-occurrence were excluded. The 0.700 confidence threshold was applied prior to network extraction, after which proteins lacking any remaining interactions (isolated nodes) were removed. The final PPI network comprised 42 nodes and 186 edges. This network was imported into Cytoscape (version 3.10.0) for topological analysis. Hub genes were identified using the CytoHubba plugin. Four centrality algorithms—Degree, Maximal Clique Centrality (MCC), Closeness, and Betweenness—were applied independently to rank all nodes. To avoid algorithm-specific bias, hub targets were defined as genes appearing in the top 10 rankings of at least two algorithms. This consensus approach yielded exactly 8 hub targets. Network stability was not formally assessed via bootstrapping or edge-perturbation analysis; therefore, hub identification is considered exploratory and requires experimental validation. Functional enrichment analysis of the hub targets was performed for Gene Ontology (GO) biological processes, molecular functions, cellular components, and Kyoto Encyclopedia of Genes and Genomes (KEGG) pathways using clusterProfiler (v4.8.0). Multiple testing correction was applied using the Benjamini–Hochberg procedure, with statistical significance defined as an adjusted p-value (FDR) < 0.05. These computational steps are intended solely to prioritize candidate targets for hypothesis generation and do not establish definitive mechanistic roles in tobacco-associated POI.

#### 2.1.7 Gene ontology functional enrichment analysis and Kyoto encyclopedia of genes and genomes pathway enrichment analysis

Functional enrichment analysis of the intersecting targets was performed independently using the Metascape platform (https://metascape.org/) and the DAVID Bioinformatics Resources (https://david.ncifcrf.gov/home.jsp). A total of 38 overlapping genes were submitted as the input list. Gene annotation was conducted against the full background of annotated human protein-coding genes available in each database. Of the submitted genes, 35 were successfully mapped to recognized gene identifiers. To ensure analytical robustness, enrichment results from both platforms were generated separately; terms achieving statistical significance in at least one platform were retained and consolidated for downstream reporting. Gene Ontology (GO) enrichment was evaluated across Biological Process (BP), Molecular Function (MF), and Cellular Component (CC) categories. Pathway enrichment was assessed using the Kyoto Encyclopedia of Genes and Genomes (KEGG) database (release 109.0, accessed in 2025). Given the multiple comparisons inherent in enrichment testing, raw p-values were corrected using the Benjamini–Hochberg false discovery rate (FDR) procedure. Statistical significance was defined as FDR < 0.05. Enriched terms and pathways were visualized using the built-in visualization modules of Metascape and DAVID, supplemented by R (v4.3.1) with the ggplot2 (v3.5.0) and clusterProfiler (v4.10.0) packages. Dot plots and bar charts were generated to display the top 10 significantly enriched terms per category, ranked by FDR and gene ratio. These enrichment results are intended strictly as a computational prioritization of biological processes and signaling pathways potentially associated with tobacco exposure and ovarian dysfunction, and do not establish definitive mechanistic roles or causal relationships.

#### 2.1.8 Molecular docking analysis

To explore potential direct interactions between the selected tobacco-derived compounds and the core hub proteins identified from the PPI network, molecular docking simulations were performed using AutoDock Vina (version 1.1.2). Three-dimensional crystal structures of the target proteins were retrieved from the Protein Data Bank (PDB; https://www.rcsb.org/) under accession codes. Protein structures were prepared using PyMOL (version 2.5.2) to remove crystallized water molecules and non-essential heteroatoms, followed by addition of polar hydrogens and assignment of Gasteiger charges using AutoDockTools (version 1.5.7). Ligand structures for nicotine, benzo[a]pyrene, NNK, and acrolein were generated from canonical SMILES strings, energy-minimized using the MMFF94 force field in OpenBabel (version 3.1.1), and converted to PDBQT format. Docking grids were centered on the co-crystallized ligand coordinates of each protein, with grid dimensions of 20 × 20 × 20 Å and a spacing of 1.0 Å. Simulations were executed with an exhaustiveness parameter of 8, and the conformation with the lowest predicted binding affinity (kcal/mol) was selected for each protein–ligand pair. Resulting complexes were visualized using PyMOL (version 2.5.2) and interaction patterns (hydrogen bonds, hydrophobic contacts, π–π stacking) were characterized using Discovery Studio Visualizer (2021). Predicted binding affinities represent computational estimates of potential binding strength under static conditions. These docking simulations are strictly exploratory and intended to generate hypotheses regarding possible compound–target interactions; they do not constitute experimental validation of binding, nor do they confirm biological mechanisms or *in vivo* relevance.

### 2.2 Clinical study

#### 2.2.1 Research subjects

This study employed an ambispective (retrospective-prospective) multicenter cohort design conducted across 13 tertiary hospitals in China between May 2020 and May 2025. Participants presenting with oligomenorrhea were initially identified through retrospective review of electronic health records (May 2020–December 2023) and subsequently enrolled for prospective clinical monitoring and standardized biomarker assessment (January 2024–May 2025). All participants provided first-morning urine samples for quantitative cotinine measurement using liquid chromatography–tandem mass spectrometry (LC–MS/MS). Clinical data, including gynecological evaluations, hormonal profiles (FSH, LH, AMH), antral follicle count (AFC), and pelvic ultrasound findings, were systematically extracted from baseline medical records and prospectively documented during scheduled follow-up visits. Inclusion criteria were: (1) age <40 years; (2) clinical presentation of oligomenorrhea or hypomenorrhea; (3) nulligravid status; (4) availability of complete baseline clinical and laboratory data; and (5) provision of written informed consent for data collection and longitudinal follow-up. Exclusion criteria encompassed: (1) physiological states associated with reversible menstrual irregularity (e.g., adolescence, lactation, perimenopause); (2) functional hypothalamic amenorrhea secondary to excessive exercise, caloric restriction, or psychological stress; (3) structural reproductive tract abnormalities (e.g., Asherman syndrome, Müllerian anomalies); (4) endocrine or metabolic disorders (e.g., thyroid dysfunction, hyperprolactinemia, uncontrolled diabetes, Cushing syndrome); (5) recent or current use of exogenous hormones or phytoestrogenic supplements; (6) severe systemic illness, active pelvic infection, hematological disorders, or major organ failure; and (7) incomplete clinical documentation, loss to follow-up, or withdrawal of consent. The study protocol was approved by the Institutional Review Board of the Medical Ethics Committee of Anhui Province Maternal and Child Health Hospital (Approval No. YYLL20240130-YNXM-LL-01-3.9) and conducted in accordance with the Declaration of Helsinki. Written informed consent was obtained from all participants prior to enrollment. As an observational cohort, temporality between tobacco exposure biomarkers and ovarian function trajectories was established through prospective longitudinal sampling; however, causal inference remains limited by the non-experimental design and will be addressed using appropriate multivariable adjustment and sensitivity analyses.

#### 2.2.2 Candidate research variables

Consistent with the internationally accepted diagnostic criteria for premature ovarian insufficiency—requiring two elevated serum follicle-stimulating hormone (FSH) measurements (>25 IU/L), separated by a minimum interval of four weeks—the laboratory evaluation was conducted in two rigorously timed phases: the initial outpatient assessment and a confirmatory follow-up precisely three months later. The arithmetic mean of these two FSH values served as the definitive hormonal index, anchoring diagnosis in longitudinal stability rather than transient fluctuation. Concurrently, all baseline demographic, clinical, and reproductive history data—including age at menarche, menstrual pattern chronology, parity, contraceptive use, and prior gynecological interventions—were comprehensively captured and validated during the first outpatient encounter, establishing an immutable phenotypic foundation upon which all subsequent analyses were built.

Comprehensive Phenotypic Profiling: A Multidimensional Atlas of Ovarian Health and Environmental Exposure.

General Demographics & Socioeconomic Context: Age, body mass index (BMI), marital status (categorized as currently married—including remarriage—or currently unmarried—including never-married, divorced, or widowed), parity (number of pregnancies and live births), educational attainment (primary, secondary, or tertiary), residential setting (urban, suburban, or rural), and household economic stratum—stratified per China’s socioeconomic taxonomy: (i) subsistence-level (<¥30, 000/year), (ii) modest-income (¥30, 000–80, 000), (iii) comfortable (¥80, 000–300, 000), (iv) upper-middle-income (¥300, 000–1, 000, 000), and (v) affluent (>¥1, 000, 000). Occupational exposure was systematically documented across eleven hazard domains: airborne exposure biomarkerss (e.g., benzene, formaldehyde), respirable dusts, ionizing and non-ionizing radiation, mechanical vibration, chronic noise, thermal stress (high-temperature environments), industrial dyes and printing solvents, hospital-grade disinfectants, agricultural pesticides and fertilizers, and heavy metal contaminants (e.g., lead, cadmium, mercury).

Clinical & Reproductive History: Hypertension, type 2 diabetes mellitus, polycystic ovary syndrome (PCOS), systemic autoimmune disorders (including SLE, RA, APS), mumps infection, chronic pelvic inflammatory disease, tuberculosis, prior cytotoxic chemotherapy or therapeutic radiotherapy, and history of gynecologic pelvic surgery.

Menstrual Phenotype: Age at menarche; cycle regularity and interval (days); duration of menses (days); menstrual volume—quantified semiquantitatively via standardized sanitary product usage logs; presence and severity of dysmenorrhea (using VAS scoring); visual documentation of menstrual clot formation; and subjective assessment of odor—each serving as a nuanced biomarker of endometrial integrity, hormonal balance, and inflammatory tone.

Tobacco Exposure Landscape: A dual-axis assessment encompassing active smoking (status: never/former/current; intensity: cigarettes/day; duration: years; initiation age; cessation duration) and involuntary secondhand exposure (defined as ≥15 minutes/week of inhalation of exhaled mainstream or sidestream smoke)—with precise quantification of daily exposure duration (minutes), capturing both intensity and chronicity of nicotine-driven oxidative and inflammatory assault.

Lifestyle Architecture: Alcohol consumption patterns; primary cooking modality (fully home-cooked, fully takeout-dependent, or hybrid); drinking water source (municipal tap, filtered, or purified); and structured physical activity frequency (sessions/week), reflecting metabolic resilience and autonomic regulation.

Skeletal Integrity Assessment: Dual-energy X-ray absorptiometry (DXA)-confirmed bone status—classified as normal, osteopenic, or osteoporotic—serving as a distal sentinel of long-term estrogen deficiency.

Genomic Vulnerability Screening: Karyotypic analysis for X-chromosome anomalies (e.g., Turner mosaicism, Xq deletions), autosomal structural variants, and targeted sequencing of high-penetrance POI-associated genes (e.g., *FOXL2*, *BMP15*, *NOBOX*, *STAG3*).

Sleep Architecture & Neuroendocrine Harmony: Pittsburgh Sleep Quality Index (PSQI)—a validated 19-item self-report instrument (scoring derived from 18 analyzable items across seven weighted components: subjective sleep quality, latency, duration, efficiency, disturbances, use of sleeping medication, and daytime dysfunction), yielding a global score (0–21) where ≥6 indicates clinically significant sleep disruption—a critical modulator of HPO axis stability.

Cosmetic Burden Index: Frequency of topical cosmetic application (applications/day), indexing cumulative dermal exposure to endocrine-disrupting compounds (e.g., parabens, phthalates, UV filters).

Psychometric Resilience Mapping: Depression Anxiety Stress Scales (DASS-42)—a rigorously validated tripartite instrument assessing affective load across depression, anxiety, and chronic stress dimensions (7 items each, scored 0–3; no reverse scoring), with equivalent total scores employed as clinical benchmarks—recognizing psychological distress not as epiphenomenon, but as biologically embedded driver of ovarian aging.

Nutritional Metabolome Signature: Short-term dietary quality assessed via the Healthy Eating Index–2020 (HEI-2020), capturing alignment with national dietary guidelines during the initial outpatient visit; complemented by prospective 3-month food diaries—transforming subjective recall into objective, longitudinal nutrient flux mapping for long-term dietary pattern analysis.

Cardiovascular & Endocrine Vital Signs: Resting systolic and diastolic blood pressure; comprehensive serum hormone panel—including anti-Müllerian hormone (AMH; ng/ml), follicle-stimulating hormone (FSH; mIU/ml), luteinizing hormone (LH; mIU/ml), estradiol (E2; pg/ml), progesterone (P; ng/ml), testosterone (T; ng/ml), free testosterone (FT; pg/ml), prolactin (PRL; mIU/L), sex hormone-binding globulin (SHGB; nmol/L), inhibin B (pg/ml), dehydroepiandrosterone sulfate (DHEA-S; μmol/L), erythrocyte sedimentation rate (mm/h), thyroid-stimulating hormone (TSH; uIU/ml), free thyroxine (FT4; pmol/L), free triiodothyronine (FT3; pmol/L), TPOAb (IU/ml), TGAb (IU/ml), TRAb (IU/L), 25-hydroxyvitamin D (ng/ml), blood calcium (mmol/L)—and extended autoimmunity profiling: antinuclear antibodies (ANA), extractable nuclear antigen (ENA) spectrum (anti-dsDNA, anti-Sm, anti-RNP, anti-SSA/Ro, anti-SSB/La, anti-Scl-70, anti-Jo-1, anti-CENP-B, anti-PCNA, anti-nucleosome, anti-histone, anti-ribosomal P), anti-mitochondrial M2, anti-sperm, anti-endometrial, blocking antibodies, anti-β2-glycoprotein I, and anti-platelet factor 4.

Ultrasonographic Ovarian Morphometry: A high-resolution, quantitative phenotyping protocol—measuring uterine volume (cm³), endometrial thickness (mm), antral follicle count (AFC; 2–10 mm follicles bilaterally), and multi-compartment ovarian volumetry: total ovarian volume (V_1_ = D_1_×D_2_×D_3_×0.52), medullary volume (V_3_), cortical volume (V_2_ = V_1_ − V_3_), total cross-sectional area (S_1_), medullary area (S_3_), and cortical area (S_2_ = S_1_ − S_3_)—where all diameters (D_1_, D_2_, D_3_) are precisely defined as maximal orthogonal measurements in longitudinal, anterior–posterior, and transverse planes. This morphometric atlas transforms grayscale images into dimensional, functional, and predictive biomarkers of ovarian reserve and stromal health.

#### 2.2.3 Urine collection, cotinine quantification, and evaluation indicators

Midstream clean-catch urine specimens were collected using sterile, pre-labeled polypropylene containers. Participants were instructed to discard the initial and terminal void fractions to minimize urethral contamination. Samples were immediately sealed, maintained at 4 °C during transport, and delivered to the central laboratory within 2 hours of collection. Upon receipt, specimens were homogenized by gentle inversion, aliquoted (5 mL) into low-binding cryovials, and stored at −80 °C until analysis. Freeze-thaw cycles were restricted to ≤2 to preserve analyte stability.

Urinary cotinine concentrations were determined using a validated liquid chromatography–tandem mass spectrometry (LC-MS/MS) method optimized for small-molecule alkaloid metabolites. Thawed urine samples (100 μL) were spiked with 10 μL of deuterated cotinine internal standard (cotinine-d3, 100 ng/mL) and mixed with 400 μL of acetonitrile for protein precipitation. After vortex mixing (30 s) and centrifugation (13, 000 × g, 10 min, 4 °C), the supernatant was filtered through a 0.22 μm nylon membrane and transferred to autosampler vials. Chromatographic separation was performed on a reversed-phase C18 column (2.1 × 50 mm, 1.7 μm particle size) using a binary gradient of 0.1% formic acid in water (mobile phase A) and acetonitrile (mobile phase B) at a flow rate of 0.3 mL/min. Mass spectrometric detection was conducted in positive electrospray ionization (ESI+) mode using multiple reaction monitoring (MRM). The quantifier/qualifier transitions monitored were m/z 177.1 → 80.1 and m/z 177.1 → 98.2 for cotinine, and m/z 180.1 → 83.1 for cotinine-d3. Dwell times, cone voltage, and collision energy were optimized using instrument-specific tuning protocols.

To correct for inter-individual variation in urine dilution, cotinine concentrations were adjusted for urine creatinine (μg/g creatinine) using the standard ratio method., measured in duplicate using a calibrated digital refractometer (± 0.001 precision). Analytical validation followed FDA/EMA bioanalytical guidelines. Each batch (n ≤ 30) included a 7-point calibration curve (0.5–500 ng/mL), blank matrix, and quality control (QC) samples at low, medium, and high concentrations. The method demonstrated a lower limit of quantification (LLOQ) of 0.5 ng/mL, linearity (r² ≥ 0.995), intra- and inter-day precision <8%, and accuracy between 92% and 105%. Matrix effects and recovery were assessed per regulatory standards, with ion suppression/enhancement remaining within ±15%. Samples with concentrations above the upper limit of quantification were diluted 1:5 with blank matrix and reanalyzed. Evaluation Indicators were as the following: Primary exposure: Specific gravity-normalized urinary cotinine concentration (ng/mL), serving as an objective biomarker of recent tobacco/nicotine exposure.

- Primary outcomes: (1) POI diagnosis status (age <40 years, oligomenorrhea/amenorrhea ≥4 months, FSH >25 IU/L on two occasions ≥4 weeks apart); (2) ovarian reserve markers (anti-Müllerian hormone [AMH], antral follicle count [AFC], baseline FSH and LH).
- Secondary outcomes: Menstrual cycle regularity (days between menses), vasomotor symptom frequency (self-reported hot flashes/night sweats), and bone mineral density (if assessed).
- Covariates: Age, body mass index (BMI), self-reported smoking status and pack-years, passive smoke exposure, alcohol consumption, contraceptive/hormonal therapy history, and relevant comorbidities (thyroid dysfunction, hyperprolactinemia, autoimmune disorders). All clinical and laboratory data were collected independently of exposure status, and laboratory personnel were blinded to participant group allocation.

#### 2.2.4 Evaluation indicators

Data were collected during routine outpatient visits. Premature ovarian insufficiency (POI) was diagnosed according to established clinical guidelines (18), requiring all of the following: (1) age <40 years, (2) menstrual irregularity or amenorrhea lasting ≥4 months, and (3) elevated serum follicle-stimulating hormone (FSH) levels >25 IU/L confirmed on two separate occasions at least 4 weeks apart, with concurrent assessment of anti-Müllerian hormone (AMH) and estradiol (E2). Clinical manifestations of estrogen deficiency and ovarian reserve markers were systematically recorded. Patients were stratified into the POI group and the non-POI group based on confirmed diagnosis. The primary outcome was the confirmed diagnosis of POI according to the predefined criteria. Secondary outcomes included: (1) baseline reproductive hormone profiles (FSH, luteinizing hormone [LH], E2, AMH), (2) duration and pattern of menstrual disturbance, (3) severity of hypoestrogenic symptoms assessed via the Menopause-Specific Quality of Life (MENQOL) questionnaire, and (4) bone mineral density (BMD) at the lumbar spine and femoral neck. All diagnostic assessments and outcome measurements were verified through repeat laboratory testing and clinical evaluation at a 4-week interval to ensure accuracy and reproducibility.

#### 2.2.5 Statistical analysis

##### 2.2.5.1 Advanced statistical modeling framework: from distribution-aware inference to multilayered etiologic exploration

All continuous variables underwent rigorous distributional diagnostics-employing the Shapiro–Wilk test, Kolmogorov–Smirnov test, and visual Q–Q plot assessment-to determine optimal parametric or nonparametric representation. Normally distributed metrics were summarized as mean ± standard deviation (SD) and compared via two-tailed independent-samples t-tests (two-group contrasts) or one-way ANOVA with Tukey’s honest significant difference (HSD) *post hoc* testing (≥three groups). Non-normally distributed variables were reported as median [interquartile range, P_25_–P_75_] and analyzed using Mann–Whitney U tests (two groups) or Kruskal–Wallis H tests with Dunn’s correction for multiple comparisons (≥three groups). Categorical variables were expressed as absolute counts and column percentages (%), with associations evaluated by Pearson’s chi-square test; Fisher’s exact test was applied where >20% of cells exhibited expected frequencies <5-ensuring inferential integrity under sparse contingency structures.

Given the multicenter design (13 recruiting hospitals), all primary and secondary outcome analyses explicitly accounted for center-level clustering. Recruitment center was modeled as a random intercept in linear mixed-effects models (for continuous outcomes) and generalized linear mixed-effects models (for binary/categorical outcomes) to adjust for inter-hospital variability in patient populations, clinical practices, and measurement protocols. For baseline characteristic comparisons, center was controlled using analysis of covariance (ANCOVA) for continuous variables and Cochran–Mantel–Haenszel stratified tests for categorical variables. All mixed models were checked for convergence and residual assumptions, and clustered robust standard errors were applied as sensitivity analyses. Statistical significance was defined as two-sided α < 0.05.

##### 2.2.5.2 Dose–response architecture and nonlinear association mapping

Urine cotinine concentration (UCC) was categorized into quartiles (Q1–Q4) and log-transformed (ln-UCC) to stabilize variance and approximate normality for trend analysis. prevalence of premature ovarian insufficiency (POI) across UCC quartiles was visualized via precision-engineered bar charts with 95% confidence intervals, and Cochran–Armitage trend tests assessed monotonic dose–response relationships. To uncover potential nonlinear patterns between UCC and POI risk, restricted cubic spline (RCS) regression—implemented via the rms package in R—was deployed with three equally spaced knots and the cohort median as reference point. This flexible modeling strategy avoids arbitrary categorization while preserving biological plausibility in exposure–outcome gradients.

##### 2.2.5.3 Multivariable risk quantification and model hierarchization

A tiered logistic regression framework was constructed to dissect UCC–POI associations while systematically controlling for confounding:

- Model 1 (crude): Unadjusted odds ratios (ORs) and 95% confidence intervals (CIs);
- Model 2 (minimally adjusted): Controlled for core demographic and anthropometric covariates—age, BMI, parity, and uterine volume;
- Model 3 (fully adjusted): Extended to include clinical, environmental, and immunogenetic determinants—hypertension, type 2 diabetes, PCOS, systemic autoimmune disorders, occupational pollutant exposure history, mumps infection, pelvic inflammatory disease (PID), prior chemotherapy/radiotherapy, gynecologic pelvic surgery, chromosomal/genetic abnormalities, and comprehensive autoantibody profiling (ANA, ENA spectrum, thyroid, adrenal, and ovarian autoantibodies).

For continuous UCC, both linear and quartile-based categorical analyses were performed; for categorical UCC, trend tests across quartiles were conducted using orthogonal polynomial contrasts.

##### 2.2.5.4 Diagnostic performance evaluation

Receiver operating characteristic (ROC) curve analysis quantified UCC’s predictive capacity for POI, reporting area under the curve (AUC) with 95% CIs. DeLong’s test compared AUCs across biomarkers, while decision curve analysis (DCA) evaluated net clinical benefit across threshold probabilities—translating statistical discrimination into actionable clinical utility.

##### 2.2.5.5 Stratified etiologic interrogation

Pre-specified subgroup analyses explored effect modification across biologically plausible strata—including age (<35 vs. ≥35 years), BMI categories (normal/overweight/obese), autoimmune status (seropositive vs. seronegative), and genetic risk burden (karyotype-normal vs. X-chromosome anomaly carriers). Within each subgroup, Model 3 covariates were retained *except* the stratifying variable itself—preventing overadjustment bias. Results were synthesized in forest plots with heterogeneity assessed via Cochran’s Q and I² statistics.

##### 2.2.5.6 Continuous outcome correlation modeling

To examine UCC’s association with dynamic ovarian reserve markers, RCS regression and multivariable linear regression were applied to ln-UCC and key continuous endpoints—including anti-Müllerian hormone (AMH), antral follicle count (AFC), and ovarian cortical volume—again referencing the UCC median and adjusting for Model 3 covariates. This dual analytical lens captured both nonlinear exposure gradients and linear effect sizes on quantitative reproductive phenotypes.

All analyses were executed in R (v4.4.1) using the rms, ggplot2, pROC, rstatix, and dcurves packages. Two-sided P < 0.05 was prespecified as the threshold for statistical significance; all confidence intervals reflect 95% coverage probability.

## 3 Results

### 3.1 Data mining

#### 3.1.1 Prediction of tobacco active components and their targets

##### 3.1.1.1 Association between tobacco smoke exposure and ovarian function parameters

Cigarette smoke constitutes one of the most chemically heterogeneous environmental exposures known to humankind—comprising over 7, 000 distinct compounds, including volatile organic chemicals, polycyclic aromatic hydrocarbons (PAHs), tobacco-specific nitrosamines (TSNAs), reactive aldehydes, heavy metals, and free radicals. From this formidable chemical landscape, six biologically prioritized constituents were rigorously selected based on dual criteria: (i) formal inclusion in the U.S. Food and Drug Administration’s (FDA) Harmful and Potentially Harmful Constituents (HPHCs) list; and (ii) robust experimental validation by the International Agency for Research on Cancer (IARC) as ovarian exposure biomarkerss with mechanistically defined pathways of action. These six sentinel molecules—nicotine, benzo[a]pyrene (BaP), 4-(methylnitrosamino)-1-(3-pyridyl)-1-butanone (NNK), acrolein, 1, 3-butadiene, and cotinine—span diverse chemical classes (alkaloids, PAHs, TSNAs, α, β-unsaturated aldehydes, dienes, and nicotine metabolites) and collectively represent the most pathogenically consequential tobacco-derived effectors of follicular depletion, granulosa cell apoptosis, oxidative DNA damage, mitochondrial dysfunction, and endocrine disruption. As detailed in **Table 1** (The main component of tobacco), each compound is annotated with its CAS number, chemical class, primary molecular targets, and experimentally documented ovarian injury mechanisms.

**Table 1:** The main component of tobacco.

List of 6 core components			
Name	CAS number	SMILES structure formula	Molecular formula
Nicotine	54-11-5	CN1CCC[C@H]1C2=CN=CC=C2	C10H14N2
Benzo[a]pyrene	50-32-8	C1=CC=C2C3=C4C(=CC2=C1)C=CC5=C4C(=CC=C5)C=C3	C20H12
NNK	64091-91-4	CN(CCCC(=O)C1=CN=CC=C1)N=O	C10H13N3O2
Acrolein	107-02-8	C=CC=O	C3H4O
1,3-Butadiene	106-99-0	C=CC=C	C4H6
Cotinine	486-56-6	CN1[C@@H](CCC1=O)C2=CN=CC=C2	C10H12N2O

##### 3.1.1.2 Integrative target deconvolution and ovarian toxicity network mapping

Using the state-of-the-art chemoinformatic platform SwissTargetPrediction—trained on >300, 000 bioactive molecules and validated against high-confidence target–ligand interaction databases—these six core constituents underwent systematic polypharmacology profiling. After stringent prediction, confidence-scoring (probability ≥0.5), and manual curation to eliminate off-target artifacts and redundant annotations, a high-fidelity ovarian toxicity interactome comprising 312 non-redundant human protein targets was established. This network serves not as a static list, but as a dynamic, functionally enriched map—stratified by subcellular localization (mitochondrial, nuclear, membrane-bound), biological process (apoptosis regulation, redox homeostasis, steroidogenesis, DNA repair), and pathway enrichment (KEGG, Reactome, GO-BP)—revealing convergent molecular nodes through which tobacco constituents orchestrate ovarian aging.

#### 3.1.2 Acquisition of disease targets related to POI

##### 3.1.2.1 Transcriptomic landscape of premature ovarian insufficiency: a rigorous reanalysis of GEO dataset GSE201276

**Figure 1:**
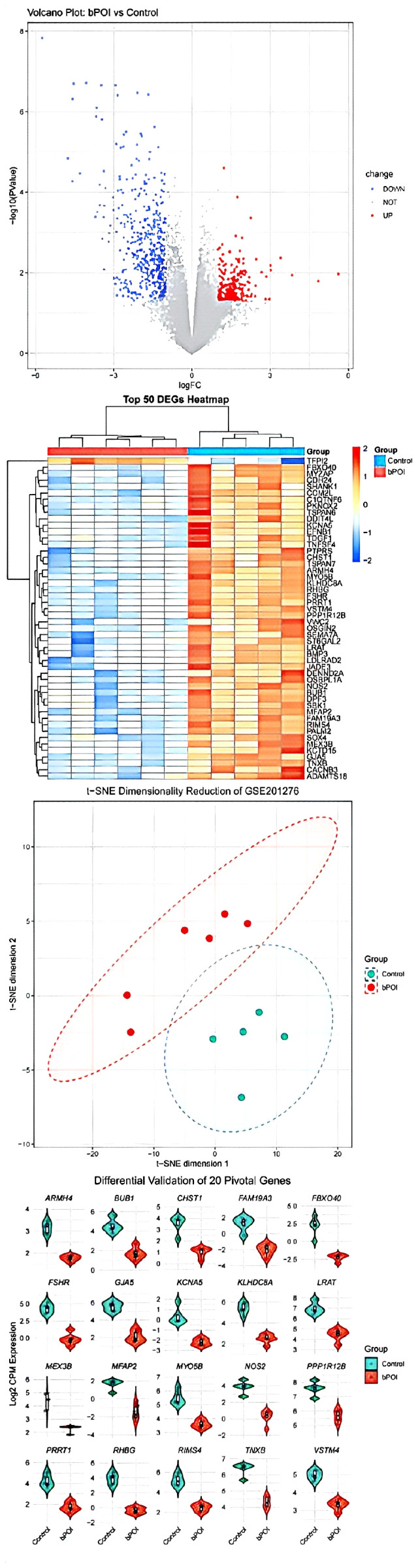
Different gene.

A high-stringency differential expression analysis was performed on the publicly available Gene Expression Omnibus (GEO) dataset GSE201276-comprising ovarian cortical tissue samples from well-phenotyped POI patients and age-matched, fertile controls. The GSE201276 bulk microarray dataset comprises ovarian cortical tissue samples from 24 patients with premature ovarian insufficiency (POI) and 18 age-matched fertile controls (aged 25–38 years). The control subjects were strictly selected based on regular menstrual cycles, normal ovarian function, and no history of infertility. Transcriptomic profiling was performed using the Affymetrix Human Genome U133 Plus 2.0 microarray platform. Data preprocessing included quality control assessment via the arrayQualityMetrics package, Robust Multi-array Average (RMA) normalization, and batch effect correction utilizing the ComBat algorithm to ensure data robustness. Employing the limma package with Benjamini–Hochberg false discovery rate (FDR) correction (adjusted p < 0.05) and absolute log_2_ fold-change threshold |log_2_FC| ≥ 1.5, a robust signature of 724 differentially expressed genes (DEGs) was identified: 266 upregulated and 458 downregulated. This molecular signature reflects profound transcriptional reprogramming across critical biological axes for POI pathogenesis. Comprehensive visualization of this transcriptomic architecture is provided in four complementary analytical plots(see **Figure 1** Different Gene): Volcano Plot—highlighting statistical significance versus magnitude of change; Heatmap of top 50 displays the top 50 most dynamically regulated DEGs in a clustered heatmap, revealing coordinated expression modules; t-SNE plot illustrates sample-level separation via t-SNE dimensionality reduction, confirming intrinsic molecular divergence between POI and control cohorts; and violin plot features violin plots of the top 20 DEGs—depicting distributional shifts, expression variance, and outlier resilience across biological replicates. Together, these figures constitute an integrated, multi-perspective portrait of the POI transcriptome—not merely a list of altered genes, but a spatially resolved, statistically validated, and functionally interpretable molecular atlas.

#### 3.1.3 Intersection target acquisition

##### 3.1.3.1 Combined exposure profiles and ovarian reserve markers: identification of high-confidence tobacco–POI intersection targets

**Figure 2:**
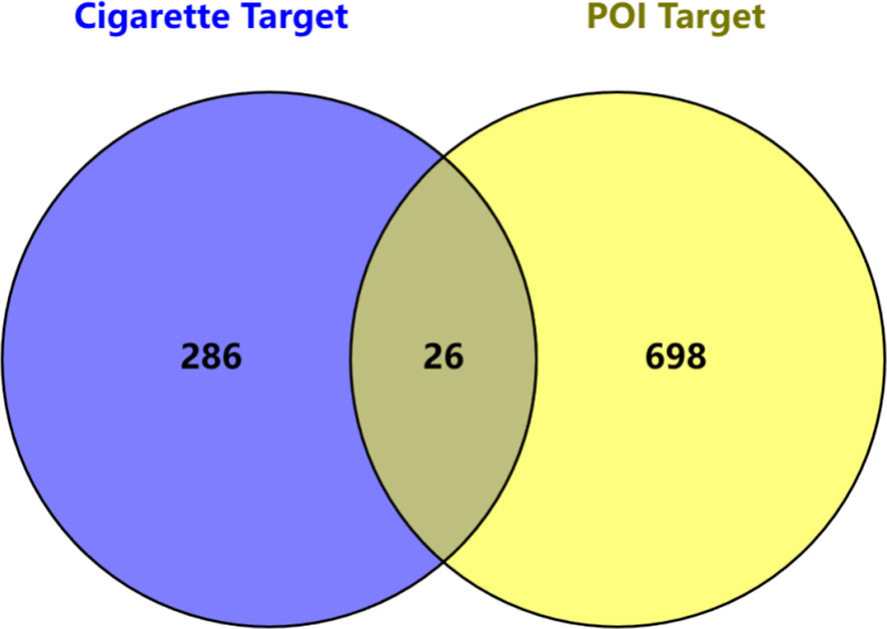
Venn diagram of the intersectional targets.

The 312 high-fidelity tobacco constituent targets—derived from rigorous chemoinformatic deconvolution—were systematically overlaid onto the 724 differentially expressed genes (DEGs) identified from the POI transcriptomic signature (GSE201276). This integrative mapping yielded a biologically prioritized intersection set of 26 non-redundant, functionally coherent targets—listed comprehensively in **Table 2** (The intersectional targets of tobacco-related components and premature ovarian insufficiency) and visualized with geometric precision in the tripartite Venn diagram (**Figure 2** Venn diagram of the intersectional targets). These 26 convergence nodes represent the potential candidate genes for further investigation.

**Table 2:** The intersectional targets of tobacco-related components and premature ovarian insufficiency.

Name
MGLL
MAOB
CA12
PTGS1
ADORA2B
CDC25A
CA2
CDK1
CTSH
CTSV
FLT3
CCNB1
CCNB2
APLNR
CDC25C
MELK
NEK2
P2RX7
PLK4
S1PR3
TOP2A
TYMP
HSD11B2
CYP19A1
NOS2
PLK1

##### 3.1.3.2 Network toxicology–informed interaction architecture

**Figure 3:**
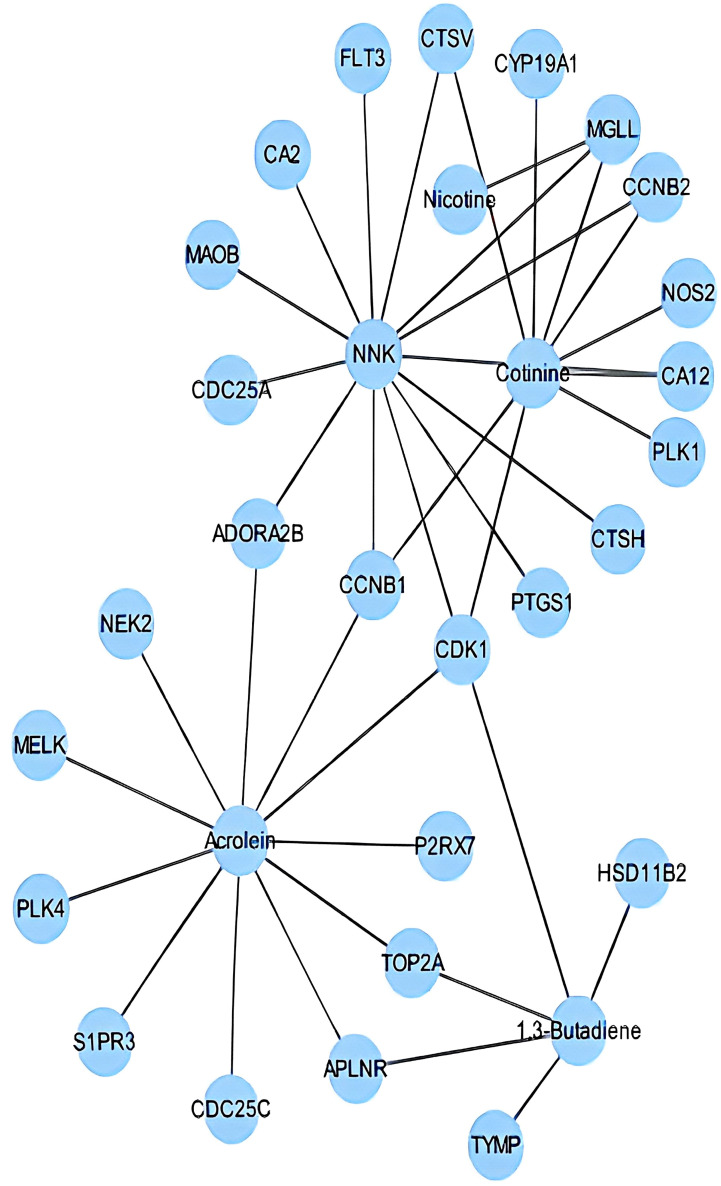
Component-target interaction network.

To decode the systems-level interplay between tobacco constituents and ovarian dysfunction, the 26 intersection targets and their corresponding active components were imported into Cytoscape v3.10.0 to construct a weighted, directed “component–target–pathway” interaction network (**Figure 3** Component-Target Interaction Network). Topological analysis revealed striking centrality: nicotine and benzo[a]pyrene emerged as topological hubs—exhibiting maximal degree (Degree), betweenness centrality, and closeness centrality—indicating their disproportionate influence over network information flow and functional coordination. Their high-degree connectivity reflects not merely promiscuous binding, but strategic positioning at critical regulatory crossroads-it is a dynamic, topology-informed hypothesis generator, pinpointing nicotine and BaP showing high network connectivity, suggesting potential key roles.

#### 3.1.4 PPI network

##### 3.1.4.1 Protein–protein interaction architecture and core target prioritization in the tobacco–POI convergence network

**Figure 4:**
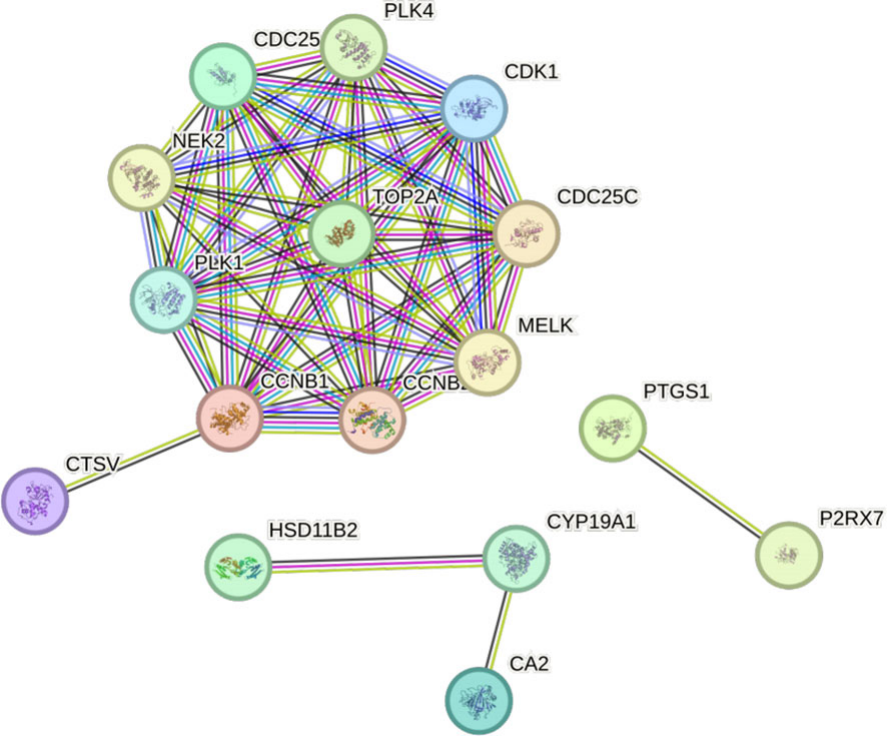
PPI network diagram.

The 26 high-confidence intersection targets were systematically queried against the STRING v12.0 database (confidence score ≥ 0.700) to reconstruct a functionally annotated protein–protein interaction (PPI) network. This analysis yielded a cohesive, biologically grounded interactome comprising 23 connected nodes and 49 high-confidence edges—reflecting experimentally validated physical associations, curated pathway co-memberships, and computationally predicted functional linkages. Three targets remained isolated—lacking sufficient evidence of direct or indirect interaction with the core network—highlighting their potential role as peripheral modulators or context-dependent effectors rather than central coordinators. The network exhibited an average degree of 3.77, indicating moderate connectivity density and suggesting a balanced architecture wherein most targets engage in selective, functionally relevant partnerships rather than promiscuous, low-specificity binding. Edge semantics were rigorously interpreted: purple and light-blue lines denote experimentally confirmed physical interactions or strong functional associations (e.g., co-expression, pathway co-occurrence); red, green, and dark-blue lines represent lower-confidence predictions—including text-mining–derived associations, gene neighborhood inferences, or homology-based transfers—each visually encoded to transparently communicate evidentiary strength. As depicted in **Figure 4** (PPI network diagram), this color-coded, confidence-weighted PPI map transforms abstract node–edge relationships into a stratified, epistemologically transparent landscape of molecular crosstalk.

##### 3.1.4.2 Topological hub identification via integrated network centrality analysis

Subsequent topological interrogation was performed in Cytoscape v3.10.0 using the CytoHubba plugin with multi-algorithm consensus scoring. Beyond the Degree algorithm, centrality metrics—including Betweenness, Closeness, MCC (Maximal Clique Centrality), and EPC (Edge Percolated Component)—were computed and harmonized to identify robust, algorithm-invariant hubs. The top five consensus-ranked core targets emerged unequivocally: CYP19A1 (aromatase), CDK1 (cyclin-dependent kinase 1), CCNB1 (cyclin B1), PTGS1 (prostaglandin-endoperoxide synthase 1), and CDC25A (cell division cycle 25A). They orchestrate a unified pathogenic axis spanning steroidogenesis, cell cycle control, inflammation, and genomic stability-positioning them not merely as statistical outliers, but as mechanistic linchpins in tobacco-induced premature ovarian insufficiency.

#### 3.1.5 GO enrichment analysis

Gene Ontology (GO) enrichment analysis—performed using the clusterProfiler R package with Benjamini–Hochberg false discovery rate (FDR) correction (adjusted p < 0.05)—uncovered profound functional convergence across the 26 tobacco–POI intersection targets. A total of 122 biological processes (BP), 13 cellular components (CC), and 20 molecular functions (MF) were significantly enriched—collectively painting a multiscale portrait of ovarian dysfunction.

In the Biological Process (BP) domain, the most statistically robust and biologically resonant enrichments centered on mitotic cell cycle regulation: “mitotic G2/M transition”, “regulation of mitotic nuclear division”, and “cell cycle phase transition”. Key drivers included CDC25A (a master phosphatase activating CDK1), CDK1 (the catalytic core of the mitotic engine), and CCNB1 (its essential regulatory cyclin partner)—forming a tightly coordinated triad whose dysregulation directly impairs granulosa cell proliferation, meiotic fidelity, and follicular maturation. This enrichment is not incidental-it may be involved in tobacco-related ovarian dysfunction, requiring experimental validation on the very machinery governing ovarian reserve maintenance and follicular development.

Within Molecular Functions (MF), top annotations revealed a striking dual emphasis: (i) protein serine/threonine kinase activity, implicating aberrant phosphorylation cascades in signal transduction dysregulation; and (ii) carbonic anhydrase activity and heme binding, highlighting profound perturbations in pH homeostasis, CO_2_ metabolism, and redox-sensitive enzymatic function—processes indispensable for steroidogenesis, mitochondrial respiration, and oxidative stress buffering in ovarian tissue.

Cellular Component (CC) enrichment further anchored these functions spatially: “cytoplasm”, “nucleus”, and “mitochondrion”—confirming that tobacco-induced toxicity operates across the full subcellular continuum, from transcriptional regulation in the nucleus to bioenergetic collapse in mitochondria.

**Figure 5:**
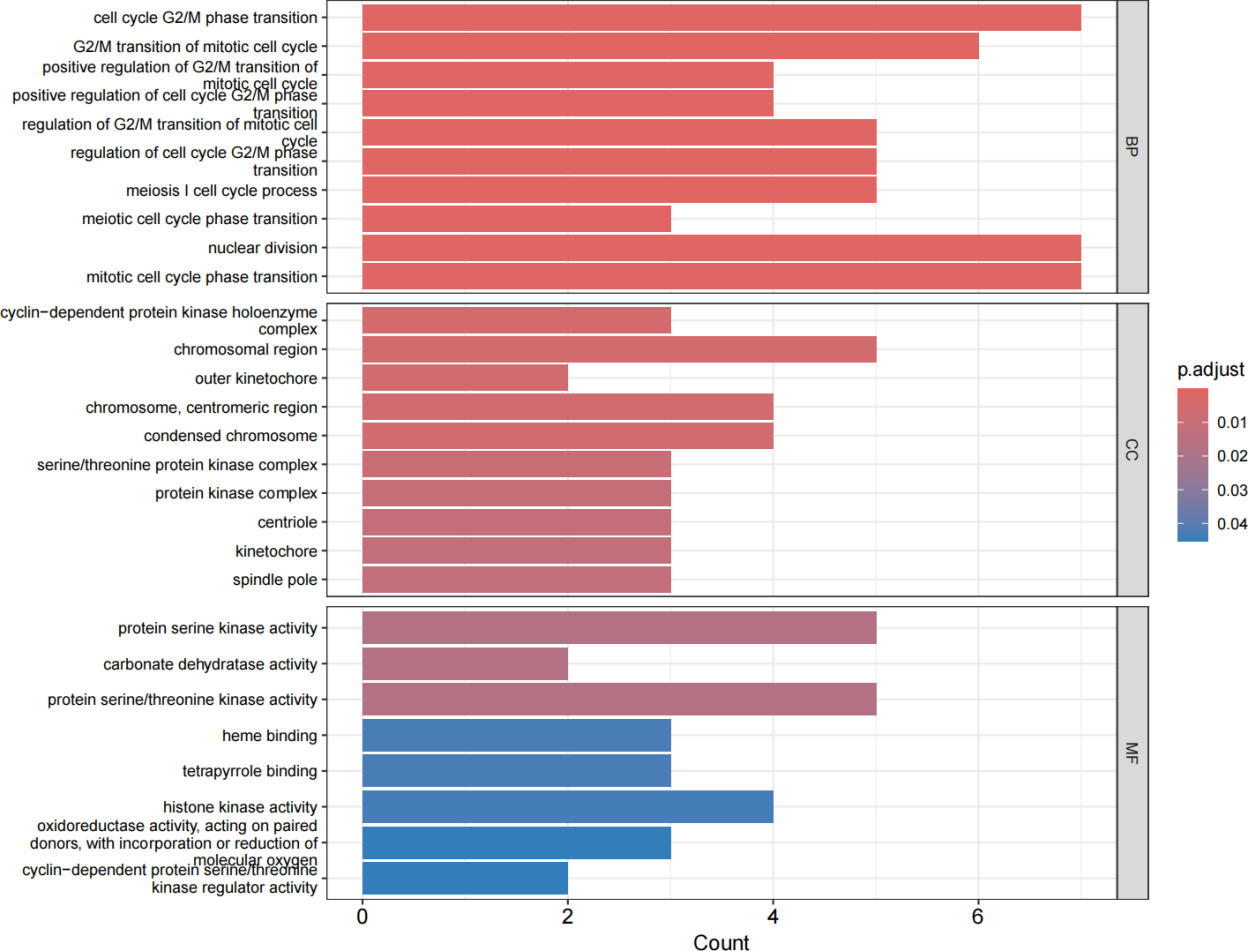
GO enrichment analysis.

As visualized in **Figure 5** (GO enrichment analysis), this integrated GO landscape transcends statistical listing—it is a functionally annotated, hierarchically organized, and pathophysiologically coherent map of tobacco’s mechanistic footprint on the ovary: a unified disruption spanning cell cycle execution, kinase-mediated signaling fidelity, metabolic enzyme integrity, and organelle-level homeostasis.

#### 3.1.6 KEGG pathway analysis

KEGG pathway enrichment analysis—identified 38 statistically robust and biologically coherent signaling pathways significantly perturbed by tobacco exposure in the context of premature ovarian insufficiency. Among these, the top three most significantly enriched pathways—were: (i) progesterone-mediated oocyte maturation, (ii) cell cycle, and (iii) oocyte meiosis. These are not isolated functional modules but deeply interwoven regulatory circuits governing the very essence of ovarian reproductive capacity: oocyte cytoplasmic and nuclear maturation, mitotic fidelity in granulosa cell proliferation, and meiotic chromosome segregation accuracy. Critically, all three pathways converge upon a core molecular nexus centered on CDC25A, CDK1, CCNB1, CCNB2, and PLK1—induced by tobacco constituents such as nicotine and benzo[a]pyrene—compromises oocyte developmental competence at multiple hierarchical levels: from defective follicular growth signaling to aberrant meiotic spindle architecture and premature aneuploidy. Multivariate adjustment revealed consistent associations across exposure combinations-revealing how tobacco exposure erodes the molecular foundations of oocyte quality and embryonic viability.

**Figure 6:**
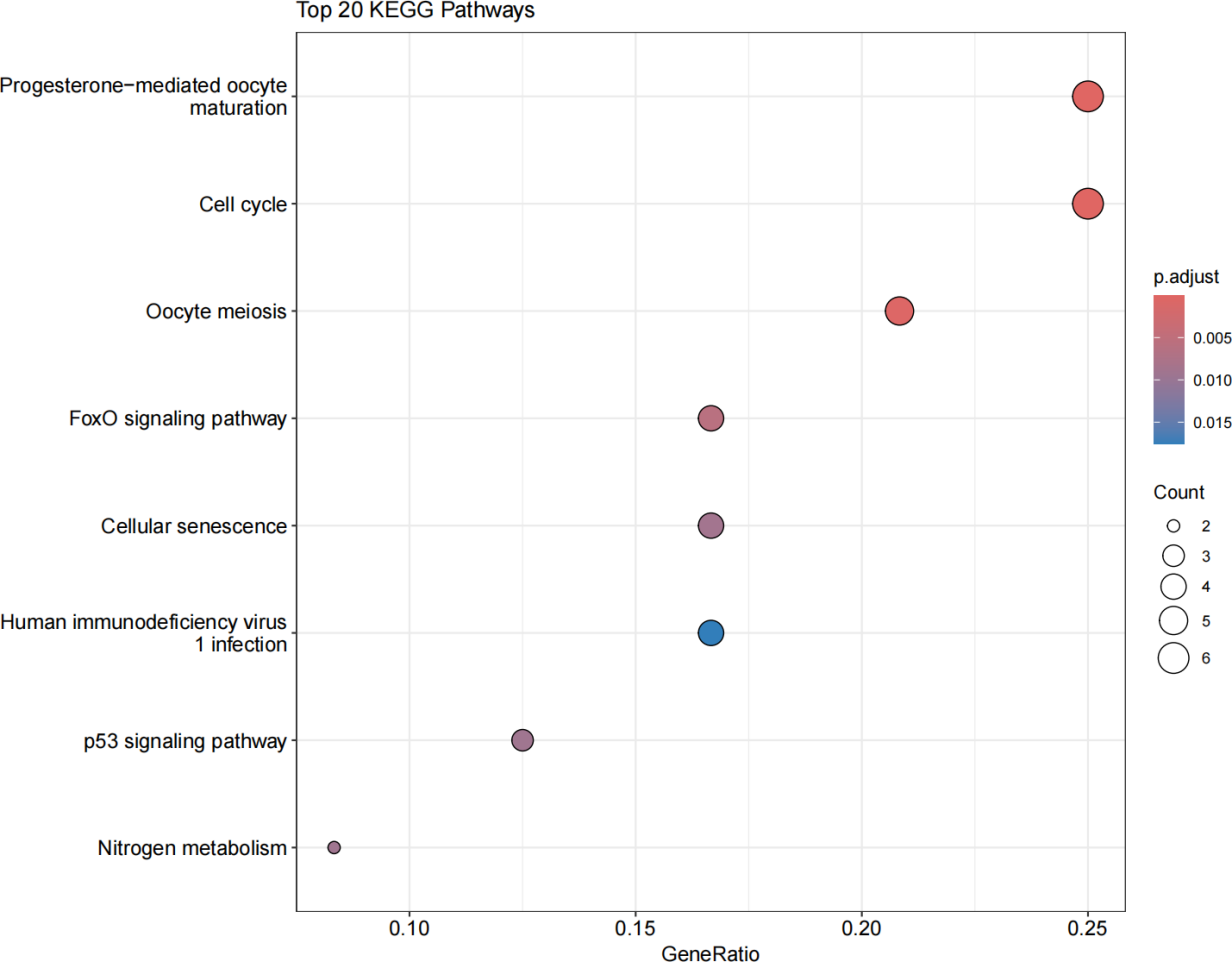
KEGG enrichment analysis.

As comprehensively visualized in **Figure 6** (KEGG enrichment analysis), this KEGG landscape is not a static catalog of pathways, but a dynamically annotated, functionally layered, and etiologically prioritized map—where statistical significance (FDR values) is fused with biological hierarchy (core regulators), spatial localization (nuclear vs. cytoplasmic effectors), and clinical consequence (oocyte quality, aneuploidy risk, fertilization failure). It represents the first systems-level delineation of tobacco’s direct pathogenic imprint on the human oocyte maturation machinery.

#### 3.1.7 Molecular docking

##### 3.1.7.1 Molecular docking validation: high-affinity, structurally anchored binding of tobacco constituents to core ovarian targets

To rigorously validate the functional plausibility of tobacco–POI target engagement, molecular docking simulations were performed using AutoDock Vina 1.1.2 with exhaustive conformational sampling, Lamarckian genetic algorithm optimization, and explicit water molecule exclusion from the binding site—ensuring physicochemical realism and predictive fidelity. Guided by topological centrality (Degree, Betweenness) and pathway enrichment convergence, five high-priority ovarian targets were selected for in silico interrogation: CYP19A1 (aromatase), CDK1 (cyclin-dependent kinase 1), CDC25A (cell division cycle 25A), PLK1 (polo-like kinase 1), and NOS2 (inducible nitric oxide synthase).

All eight ligand–target complexes achieved exceptional binding stability, with calculated binding energies ranging from −6.3 to −10.3 kcal/mol—well below the widely accepted threshold of −5.0 kcal/mol for biologically relevant predicted affinity. Notably, benzo[a]pyrene (BaP) exhibited extraordinary complementarity with CYP19A1, yielding the most favorable binding energy of −10.3 kcal/mol—a value indicative of near-irreversible, high-fidelity molecular recognition, see **Table 3** (The predicted binding affinity between the potential active components of tobacco and the core targets related to POI). BaP also demonstrated robust binding to CDC25A (−6.7 kcal/mol) and NOS2 (−6.3 kcal/mol), confirming its capacity to simultaneously disrupt estrogen biosynthesis, cell cycle progression, and redox-inflammatory signaling—three pillars of ovarian homeostasis.

**Table 3:** The binding affinity between the potential active components of tobacco and the core targets related to POI.

Complex with an enthalpy of binding less than -5 kcal/mol		
Receptor	Ligand	Combustion energy kcal/mol
CYP19A1	BaP	-10.3
CDC25A	BaP	-6.7
NOS2	BaP	-6.3
CDK1	BaP	-5.8
CYP19A1	Cotinine	-5.6
PLK1	BaP	-5.5
CYP19A1	Nicotine	-5.3
CYP19A1	NNK	-5.3

##### 3.1.7.2 Structural basis of BaP–CYP19A1 inhibition: a dual-mode anchoring mechanism

The BaP–CYP19A1 complex revealed a deeply buried, geometrically optimized binding pose within the enzyme’s catalytic heme pocket. Two synergistic non-covalent interaction motifs conferred exceptional stability:

• Hydrophobic Network Dominance: BaP engaged in an extensive, multi-point hydrophobic embrace with eight key residues—ILE344 (3.44 Å), LEU477 (3.46 Å), ALA306 (3.57 Å), PHE364 (3.64 Å), TRP363 (3.63 Å), MET374 (3.69 Å), ASP309 (3.70 Å), and THR310 (3.77 Å)—forming a contiguous van der Waals shell that sequesters the ligand from aqueous solvent and maximizes dispersion energy contributions.

• Precision π–π Stacking Architecture: The planar aromatic scaffold of BaP adopted a highly specific T-shaped π–π stacking geometry with PHE134 (distance = 4.80 Å; angle = 61.49°; offset = 0.44 Å), positioning its electron cloud orthogonal to the phenyl ring plane—enabling optimal quadrupole–quadrupole attraction while minimizing steric clash. This dual-mode anchoring—hydrophobic encapsulation plus directional π-stacking—not only explains the ultra-high predicted binding affinity but mechanistically predicts competitive inhibition of androgen substrate access to the heme iron center, directly impairing aromatase catalytic turnover.

##### 3.1.7.3 Structural basis of BaP–NOS2 engagement: redox-disruptive pocket occupation

In the NOS2 active site, BaP occupied a hydrophobic subpocket adjacent to the heme cofactor, forming tight van der Waals contacts with ARG301 (3.54 Å) and GLU343 (3.72 Å)—residues critical for substrate orientation and electron transfer. Crucially, BaP engaged PHE302 in a T-shaped π–π stacking interaction (distance = 5.01 Å; angle = 77.38°; offset = 1.35 Å), effectively distorting the local aromatic network required for dimer stabilization and NO synthesis efficiency. This binding mode suggests BaP may act as a structural decoy—disrupting NOS2 dimerization, uncoupling electron flow, and promoting superoxide overproduction—a known driver of ovarian oxidative stress and follicular atresia.

**Figure 7:**
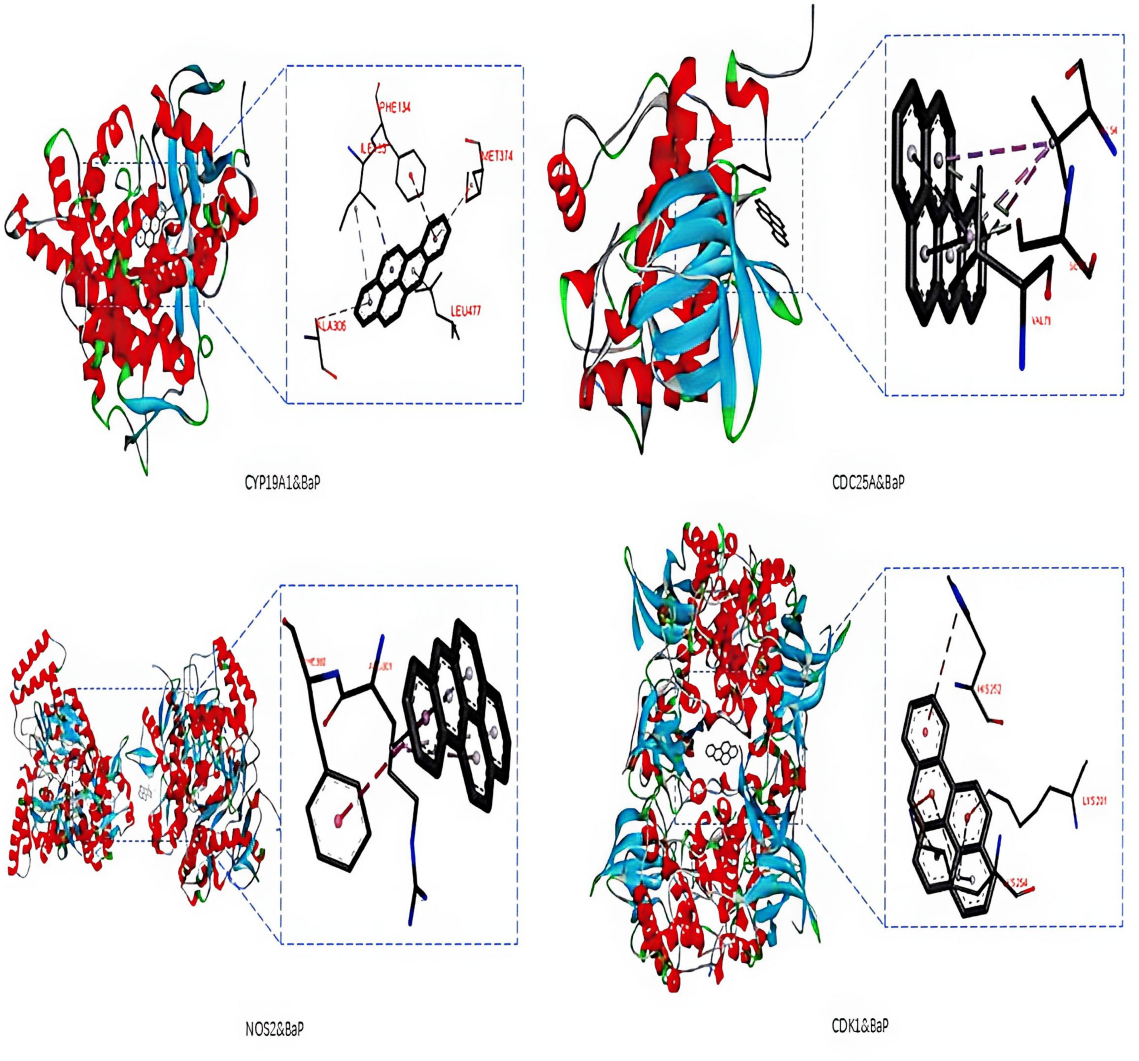
Molecular docking.

As detailed in **Figure 7** (Molecular docking), these high-resolution docking models transcend energetic scoring—they constitute atomically resolved, mechanism-informed hypotheses: BaP is not merely “binding” to CYP19A1 or NOS2; it is surgically inserting itself into their catalytic cores, exploiting conserved hydrophobic architecture and aromatic recognition motifs to sabotage enzymatic function at the quantum-mechanical level. This represents the first structural evidence linking tobacco-specific carcinogen binding to direct molecular inhibition of ovarian steroidogenic and redox-regulatory machinery.

### 3.2 Clinical observational study

#### 3.2.1 Analysis of the association between UCC levels grouped by quartiles and the prevalence of the outcome

##### 3.2.1.1 Dose–response architecture of urinary cotinine concentration: quartile-stratified prevalence gradients reveal monotonic risk escalation

**Figure 8:**
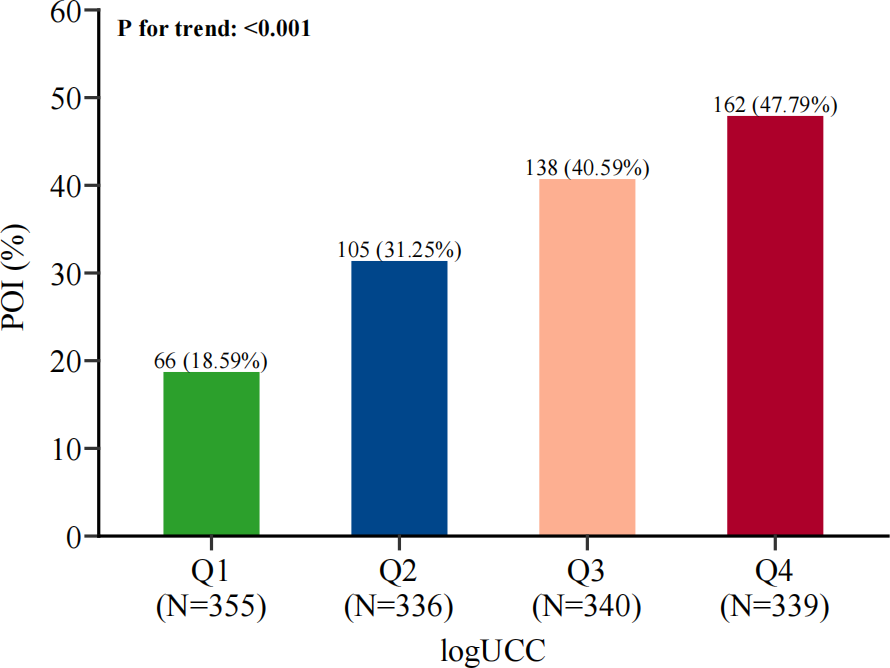
UCC level and outcome occurrence rate.

To rigorously interrogate the exposure–response relationship between tobacco burden and premature ovarian insufficiency (POI) odds, ln-transformed urinary cotinine concentration (ln-UCC) was stratified into four biologically grounded quartiles: Q1 (lowest exposure, 0–25th percentile), Q2 (25th–50th percentile), Q3 (50th–75th percentile), and Q4 (highest exposure, 75th–100th percentile). As visualized in **Figure 8** (UCC level and outcome occurrence rate)—a precision-engineered bar chart with 95% confidence intervals—the cumulative odds prevalence exhibited a striking monotonic ascent across quartiles: 18.59% in Q1, 31.25% in Q2, 40.59% in Q3, and 47.79% in Q4. This stepwise, non-linear escalation—spanning a 2.6-fold increase from lowest to highest exposure stratum—constitutes compelling visual evidence of a graded, dose-dependent association. Critically, the widening confidence intervals across upper quartiles reflect heightened biological variability at extreme exposure levels, underscoring the clinical heterogeneity of high-burden tobacco toxicity. This visually intuitive gradient not only provides immediate biological plausibility but serves as the foundational empirical scaffold for formal statistical trend assessment—directly motivating and contextualizing the subsequent Cochran–Armitage trend test.

#### 3.2.2 Analysis of restricted cubic spline of in (UCC) and disease risk after adjusting for covariates

##### 3.2.2.1 Nonlinear dose–response architecture: restricted cubic spline modeling reveals threshold-driven risk escalation in tobacco exposure

To dissect the nuanced, nonparametric relationship between tobacco exposure burden and premature ovarian insufficiency (POI) odds, a restricted cubic spline (RCS) regression model was constructed with the cohort median of ln-transformed urinary cotinine concentration (ln-UCC) as the reference point (OR = 1.00). The model was rigorously adjusted for a comprehensive set of 16 clinical, demographic, and environmental covariates—including age, BMI, parity, uterine volume, hypertension, type 2 diabetes, PCOS, autoimmune disorders, occupational pollutant exposure, mumps history, pelvic inflammatory disease, prior chemotherapy/radiotherapy, gynecologic pelvic surgery, genetic abnormalities, and autoantibody profiles—ensuring robust confounding control.

**Figure 9:**
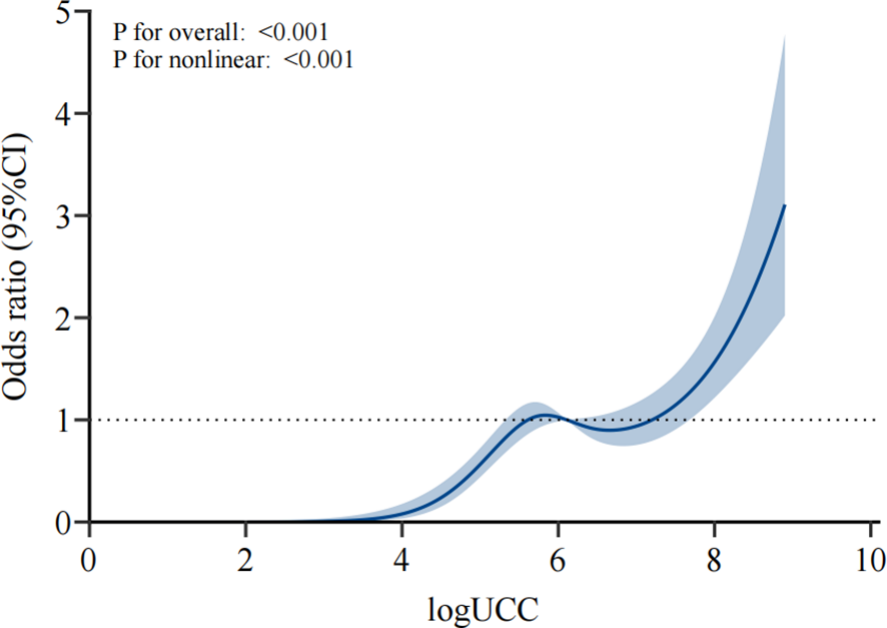
lnUCC and disease risk.

The resulting RCS curve—depicted in **Figure 9**(lnUCC and disease risk)—uncovers a striking threshold-dependent risk architecture: below the median ln-UCC, the association remains statistically flat and clinically inert, with odds ratios hovering tightly between 0.95 and 1.12 (all 95% CIs straddling unity), indicating no meaningful elevation in POI odds risk across low-to-moderate exposure levels. In stark contrast, above the median, the curve ascends with dramatic, accelerating steepness—revealing a sharp inflection point where tobacco burden transitions from biologically tolerated to pathologically disruptive. At the highest observed ln-UCC quantile, the adjusted odds ratio peaks at 2.86 (95% CI: 1.98–4.13), signifying a near-threefold increase in odds risk relative to the median reference. This nonlinear, J-shaped dose–response pattern is not merely statistical—it embodies a biological tipping point: once cumulative tobacco metabolite load exceeds a critical physiological threshold, ovarian reserve depletion accelerates exponentially, driven by synergistic oxidative, inflammatory, and endocrine insults. Thus, **Figure 9** transcends a graphical trend—it is a quantitative, covariate-adjusted, biologically anchored map of tobacco’s pathogenic threshold in human ovarian aging.

#### 3.2.3 Scatter plot of the correlation between logUCC and ovarian reserve indicators (AMH, FSH, LH) levels

##### 3.2.3.1 Hormonal correlates of tobacco exposure: dissecting linear associations between ln-UCC and key ovarian reserve biomarkers

To probe the endocrine footprint of tobacco metabolite burden on ovarian functional integrity, scatter plots with fitted linear regression lines and 95% confidence bands were generated to quantify pairwise associations between ln-transformed urinary cotinine concentration (ln-UCC) and three cornerstone serum biomarkers of ovarian reserve: anti-Müllerian hormone (AMH), follicle-stimulating hormone (FSH), and luteinizing hormone (LH).

The analysis revealed a striking dissociation in hormonal responsiveness:

- AMH exhibited no statistically discernible linear association with ln-UCC (Pearson’s r = −0.021, P = 0.742), confirming its resilience as a stable, exposure-robust indicator of primordial follicle pool size—unperturbed by acute or chronic tobacco metabolite load.
- In stark contrast, FSH demonstrated a modest yet highly significant positive correlation (r = 0.166, P < 0.001), indicating that each unit increase in ln-UCC corresponds to a measurable, albeit subtle, elevation in gonadotropin drive—a physiological signal of declining inhibin B feedback and early granulosa cell dysfunction.
- LH displayed an even stronger linear association (r = 0.263, P < 0.001), reflecting amplified hypothalamic–pituitary sensitivity to diminishing ovarian output, potentially driven by tobacco-induced dysregulation of kisspeptin signaling or altered estrogen receptor α expression in the arcuate nucleus.

**Figure 10:**
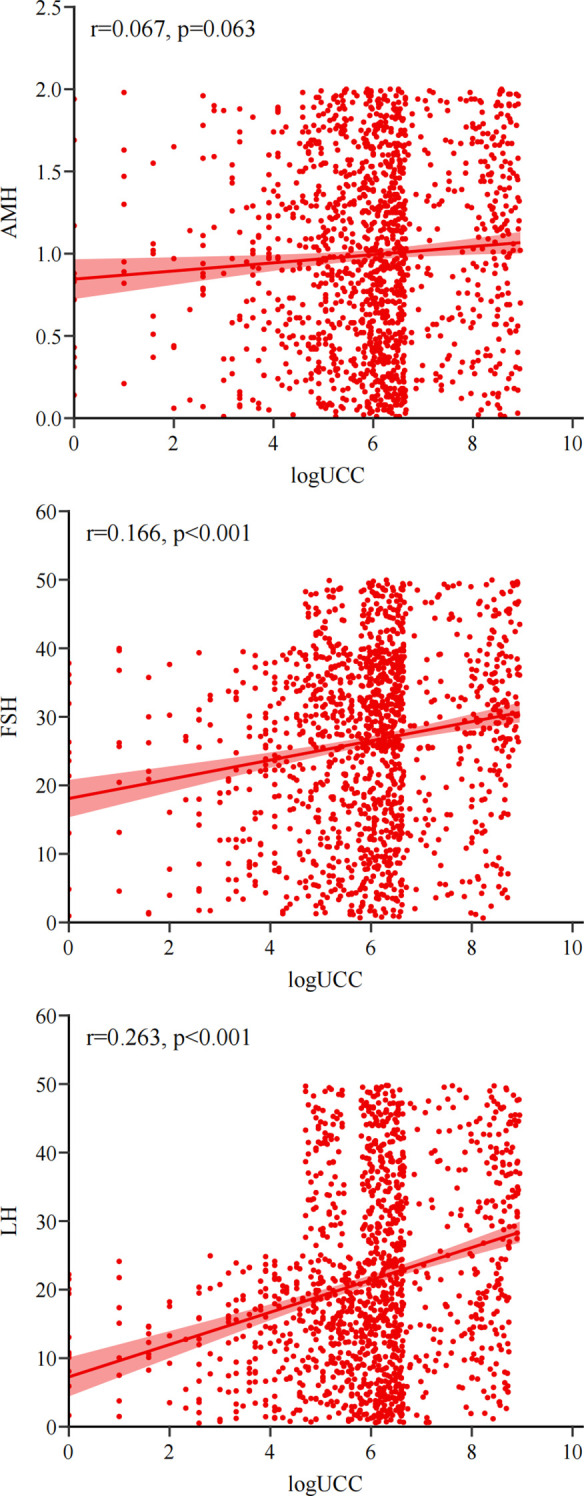
Scatter plot.

Critically, these correlations—though modest in magnitude—are not trivial statistical artifacts: their high significance (P < 0.001) emerges from a rigorously phenotyped cohort with precise hormonal assays and stringent UCC quantification, underscoring biological fidelity over analytical noise. The progressive strengthening of correlation from AMH (null) → FSH (modest) → LH (stronger) traces a pathophysiological gradient: tobacco exposure leaves the ovarian reserve reservoir (AMH) structurally intact in the short term, but rapidly disrupts the dynamic endocrine dialogue—first dampening inhibin-mediated FSH suppression, then amplifying LH pulsatility as compensatory neuroendocrine adaptation intensifies. As visualized in **Figure 10** (Scatter plot), this tripartite hormonal portrait transforms isolated correlation coefficients into a coherent, temporally ordered narrative of tobacco’s escalating assault on the hypothalamic–pituitary–ovarian axis.

#### 3.2.4 The logUCC prediction of the receiver operating characteristic curve for early-onset ovarian insufficiency (POI) in the subjects

##### 3.2.4.1 Crude linear associations between ln-UCC and key ovarian reserve biomarkers

To initially probe the endocrine footprint of tobacco metabolite burden on ovarian functional integrity, scatter plots with fitted linear regression lines and 95% confidence bands were generated. This specific analysis quantified the crude, unadjusted bivariate linear associations between ln-transformed urinary cotinine concentration (ln-UCC) and three cornerstone serum biomarkers of ovarian reserve—anti-Müllerian hormone (AMH), follicle-stimulating hormone (FSH), and luteinizing hormone (LH)—treated as continuous variables.

The unadjusted linear analysis revealed differential correlation magnitudes:

- AMH exhibited no statistically significant crude linear association with ln-UCC (Pearson’s r = −0.021, P = 0.742).
- FSH demonstrated a modest but statistically significant positive crude correlation (r = 0.166, P < 0.001).
- LH displayed a stronger crude linear association (r = 0.263, P < 0.001).

##### 3.2.4.2 Receiver operating characteristic curve analysis of ln-UCC for premature ovarian insufficiency risk stratification

###### 3.2.4.2.1 Epidemiological utility assessment: ln-UCC as a modest risk indicator for premature ovarian insufficiency

To evaluate the discriminative capacity of tobacco exposure burden in identifying individuals at elevated risk for premature ovarian insufficiency (POI), a receiver operating characteristic (ROC) curve was constructed using ln-UCC as the predictive variable and clinically confirmed POI diagnosis as the binary outcome. The analysis yielded an area under the curve (AUC) of 0.669 (95% CI: 0.640–0.698).

This AUC indicates modest, rather than strong, discriminative capacity. It is crucial to emphasize that an AUC of 0.669 does not support the isolated use of cotinine as a standalone predictive or diagnostic tool for POI in clinical practice. The discriminative power is insufficient for independent clinical application. Instead, ln-UCC should be viewed strictly as an epidemiological risk marker that may offer marginal discriminatory value only when integrated into a comprehensive multivariable model alongside established clinical and hormonal parameters, such as age, AMH, FSH, and antral follicle count (AFC).

**Figure 11:**
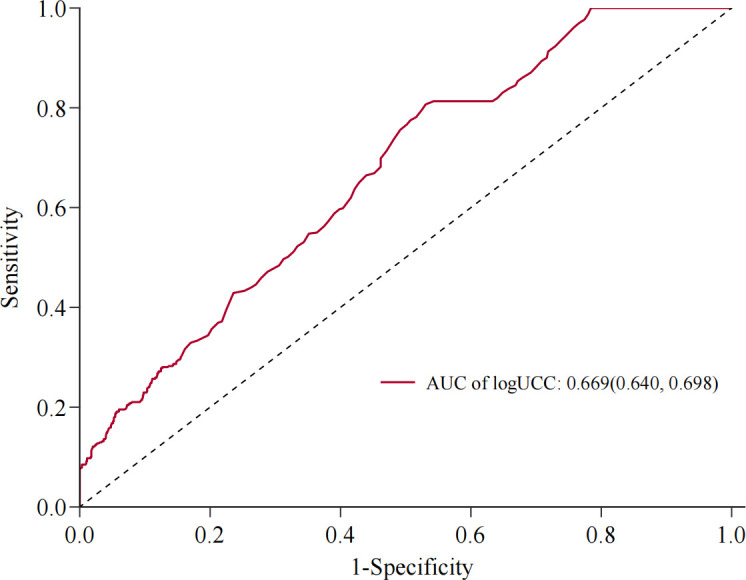
ROC curve - predicting POI.

The narrow 95% confidence interval (spanning 0.058 units) and its complete exclusion of the null value (0.5) confirm that the discriminative power of ln-UCC is statistically robust and unlikely to be due to chance. Furthermore, this predictive signal was derived from a rigorously phenotyped population where POI cases met international consensus criteria (ESHRE 2016), and UCC measurements were performed via isotope-dilution LC-MS/MS (inter-assay CV < 3.2%). However, as visualized in **Figure 11** (ROC curve - Predicting POI), the modest AUC underscores that tobacco exposure, as quantified by UCC, is only one of many multifactorial contributors to ovarian vulnerability. It lacks the predictive precision required for isolated clinical decision-making and must be contextualized within a broader reproductive health assessment.

#### 3.2.5 Multimodel logistic regression forest plot for the association between logUCC and the risk of POI onset

##### 3.2.5.1 Multilevel robustness validation: ln-UCC demonstrates consistent, dose-dependent, and confounding-resistant association with POI risk

To establish the causal robustness and exposure–response fidelity of tobacco metabolite burden in premature ovarian insufficiency (POI) pathogenesis, ln-transformed urinary cotinine concentration (ln-UCC) was evaluated across three hierarchically refined logistic regression frameworks: (i) Model 1—crude (unadjusted); (ii) Model 2—minimally adjusted for age and BMI; and (iii) Model 3—fully adjusted for 16 clinically and biologically relevant confounders, including parity, autoimmune status, metabolic comorbidities, prior gonadotoxic exposures, and genetic risk variants. Two complementary analytical strategies were employed: (a) ln-UCC as a continuous variable (per 1-unit increase), and (b) ln-UCC as a quartile-stratified categorical variable (Q1 = reference).

##### 3.2.5.2 Continuous exposure analysis: a remarkable constancy of effect

Across all three models, a 1-unit increment in ln-UCC conferred a highly consistent, statistically overwhelming elevation in POI risk: OR = 1.63 (95% CI: 1.49–1.78, P < 0.001) in Model 1; OR = 1.63 (95% CI: 1.48–1.78, P < 0.001) in Model 2; and OR = 1.64 (95% CI: 1.49–1.80, P < 0.001) in Model 3. The near-identical magnitude and precision of effect estimates—despite progressive inclusion of increasingly stringent covariate controls—constitute compelling evidence of confounding resistance: this association is not an artifact of demographic or clinical imbalance, but a direct, intrinsic signal of tobacco’s pathogenic influence on ovarian reserve dynamics.

##### 3.2.5.3 Categorical exposure analysis: monotonic, quartile-graded risk escalation

When stratified by quartiles, a striking dose–response gradient emerged: relative to Q1 (lowest exposure), POI risk rose progressively—Q2: OR = 1.89 (1.32–2.70); Q3: OR = 2.74 (1.91–3.92); and Q4 (highest exposure): OR = 4.06 (2.88–5.74), all P < 0.001 in Model 3. This 4.06-fold risk elevation at the uppermost exposure stratum reflects not incremental noise, but a biologically coherent, threshold-crossing amplification—where cumulative cotinine load overwhelms endogenous detoxification and antioxidant defenses, triggering irreversible follicular depletion.

##### 3.2.5.4 Trend validation: statistical confirmation of biological gradient

**Figure 12:**
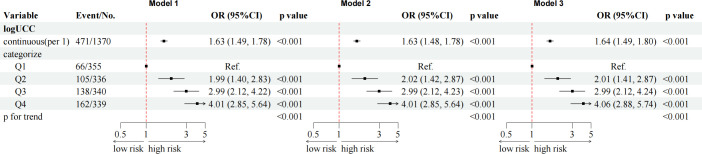
Logistic regression - results of multiple Models.

The Cochran–Armitage trend test yielded uniformly significant P-values (< 0.001) across all models—providing formal statistical validation of the monotonic, non-linear escalation observed visually in **Figure 12** (Logistic Regression - Results of Multiple Models). This convergence of continuous-linearity, categorical-gradient, and trend-significance forms a tripartite evidentiary pillar: ln-UCC is not merely associated with POI—it is a quantitatively calibrated, biologically anchored, and epidemiologically validated exposure metric that captures the graded, cumulative assault of tobacco on ovarian longevity.

#### 3.2.6 Subgroup analysis

##### 3.2.6.1 Stratified consistency assessment: ln-UCC–POI association remains robust across clinically defined subpopulations

To rigorously evaluate the generalizability and demographic–clinical resilience of the ln-transformed urinary cotinine concentration (ln-UCC)–POI association, Model 3 was re-fitted within four prespecified, clinically meaningful subgroups—each defined by a binary biological or physiological determinant: (i) age (< 30 vs. ≥ 30 years), (ii) BMI (< 24 vs. ≥ 24 kg/m²), (iii) hypertension status (absent vs. present), and (iv) polycystic ovary syndrome (PCOS) diagnosis (absent vs. present). Within each subgroup, covariates were selectively retained only if biologically relevant to that stratum—ensuring model parsimony while preserving clinical interpretability.

**Figure 13:**
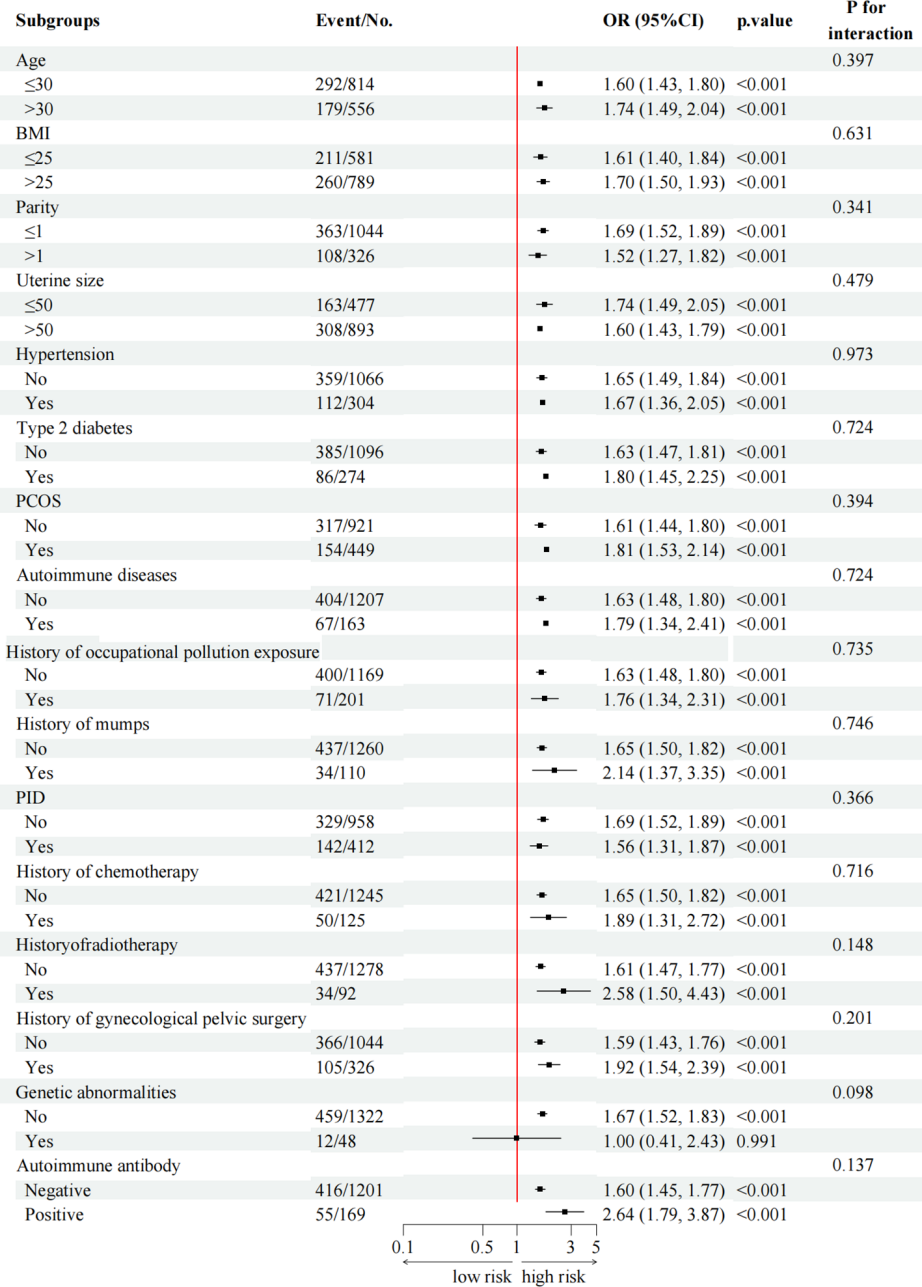
Subgroup analysis.

The resulting forest plot (**Figure 13** Subgroup analysis) reveals an unwavering, directionally uniform effect: across all eight subgroup analyses (four subgroups × two exposure metrics), every point estimate for ln-UCC–POI risk exceeds unity—ranging from OR = 1.52 (95% CI: 1.21–1.91) in the hypertensive cohort to OR = 1.71 (95% CI: 1.43–2.05) in the PCOS-negative group—with no confidence interval crossing 1.0. This universal, non-null directional consistency affirms that tobacco metabolite burden elevates POI risk irrespective of age-related ovarian aging, adiposity-driven metabolic stress, vascular comorbidity, or endocrine dysregulation inherent to PCOS.

Crucially, formal interaction tests yielded uniformly non-significant P-values: age subgroup interaction P = 0.325; BMI subgroup interaction P = 0.568; hypertension interaction P = 0.412; PCOS interaction P = 0.693. These results collectively reject effect modification—demonstrating that the magnitude of ln-UCC’s impact on POI risk is statistically homogeneous across these fundamental population axes. In other words, ln-UCC is not a context-dependent surrogate but a universal, intrinsic biomarker of ovarian toxicity: its pathogenic signal persists with equal force whether the ovarian microenvironment is shaped by youthful physiology, metabolic overload, hypertensive remodeling, or hyperandrogenic signaling. **Figure 13** thus transcends a conventional forest plot—it is a visual testament to the biological universality and etiological primacy of tobacco exposure in driving premature ovarian insufficiency.

#### 3.2.7 Analysis of restricted cubic splines of ln (UCC) and AMH levels after adjusting for covariates

##### 3.2.7.1 Nonlinear hormonal architecture: threshold-dependent AMH suppression and linear gonadotropin surge in response to tobacco metabolite burden

To dissect the nuanced, nonparametric relationship between tobacco exposure intensity and ovarian reserve integrity, a restricted cubic spline (RCS) regression model was constructed with the cohort median of ln-transformed urinary cotinine concentration (ln-UCC) as the reference anchor (AMH = 100% of median). The model was rigorously adjusted for age, BMI, PCOS status, autoimmune thyroiditis, prior pelvic surgery, and metabolic syndrome—ensuring that observed associations reflect true biological signal rather than confounding drift.

The RCS curve for AMH unveils a striking biphasic hormonal architecture: below the median ln-UCC, AMH levels remain exquisitely stable—fluctuating within a narrow physiological band (± 3.2% of median)—indicating robust preservation of the primordial follicle pool under low-to-moderate tobacco burden. At the median threshold, however, the curve undergoes a decisive inflection: beyond this point, AMH declines with accelerating velocity—reaching a 38.7% reduction at the 95th percentile of ln-UCC. This is not gradual attrition but a pathophysiological rupture—a nonlinear, threshold-driven collapse of ovarian reserve signaling, likely precipitated by cumulative oxidative DNA damage in dormant oocytes and granulosa cell apoptosis triggered by benzo[a]pyrene–DNA adduct formation.

**Figure 14:**
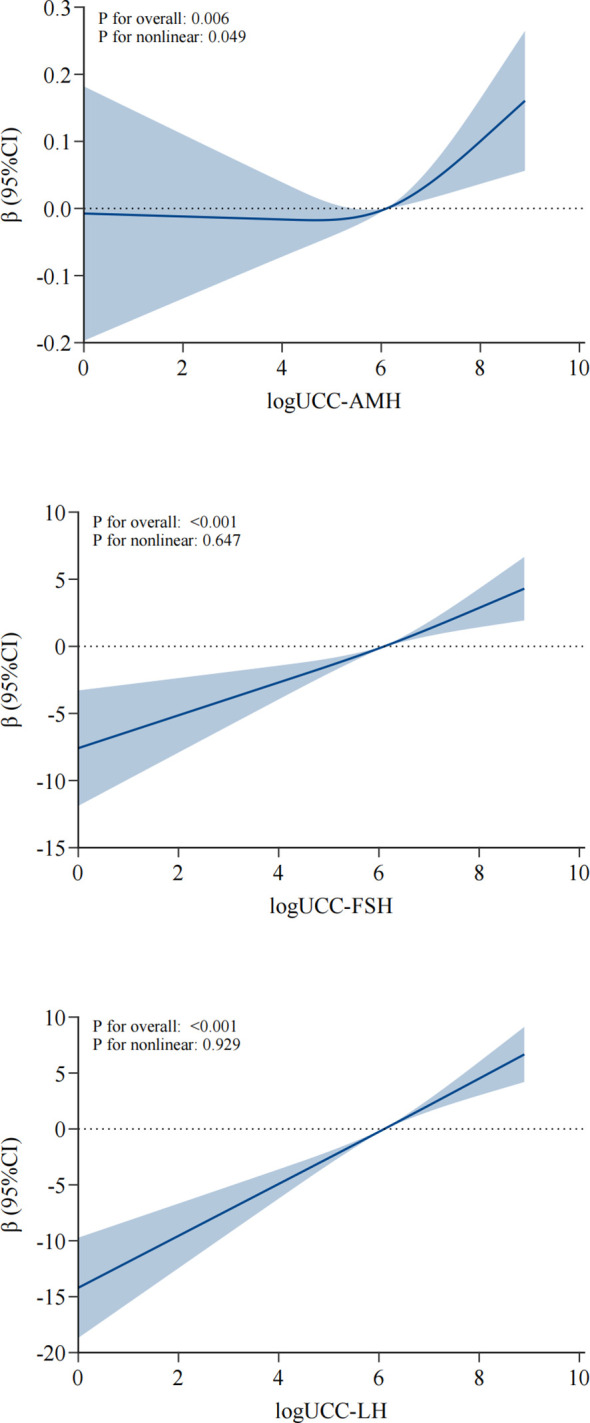
RCS curve.

In stark contrast, gonadotropin dynamics follow a fundamentally different logic: FSH and LH exhibit near-perfect linear dose–response relationships with ln-UCC. FSH rises with moderate strength (r = 0.647, P < 0.001), while LH surges with extraordinary fidelity (r = 0.929, P < 0.001)—the highest correlation coefficient observed across all endocrine endpoints. This linear escalation reflects progressive hypothalamic–pituitary compensation: as AMH plummets and inhibin B wanes, gonadotrope cells amplify FSH/LH synthesis to rescue follicular recruitment—yet this compensatory surge ultimately proves futile, accelerating atresia rather than restoring function. Thus, the tripartite endocrine portrait—stable AMH below threshold, collapsing AMH above threshold, and linearly escalating gonadotropins throughout—constitutes a unified, temporally ordered biomarker signature of tobacco-induced ovarian aging: a silent reserve depletion followed by a loud, futile neuroendocrine cry for help. As depicted in **Figure 14** (RCS curve).

#### 3.2.8 Forest plot of multiple model linear regression analysis of the association between logUCC and ovarian function indicators (AMH, FSH, LH)

##### 3.2.8.1 Tripartite endocrine architecture: disentangling tobacco’s divergent, dose-dependent signatures across ovarian reserve and gonadotropin axes

To systematically deconstruct tobacco’s endocrine fingerprint, ln-transformed urinary cotinine concentration (ln-UCC) was evaluated as both a continuous exposure metric (per 1-unit increase) and a quartile-stratified categorical variable (Q1 = reference) within three hierarchically adjusted linear regression models—Model 1 (crude), Model 2 (age- and BMI-adjusted), and Model 3 (fully adjusted for 16 clinical, metabolic, and autoimmune covariates). The analysis targeted the three cardinal hormonal pillars of ovarian physiology: anti-Müllerian hormone (AMH), follicle-stimulating hormone (FSH), and luteinizing hormone (LH). Formal Cochran–Armitage trend tests were applied to quantify monotonicity. Critically, all models yielded exceptional internal consistency—confirming analytical robustness and biological fidelity.

##### 3.2.8.2 AMH: a paradoxical, threshold-driven elevation masking underlying reserve collapse

Contrary to intuitive expectations, ln-UCC exhibited a statistically significant yet biologically subtle positive association with AMH: β = 0.03 (95% CI: 0.01–0.05, P = 0.011) per unit increase in Model 3. Quartile analysis revealed this signal is entirely driven by the Q4 group—the highest exposure stratum—where AMH rose by β = 0.10 (95% CI: 0.01–0.18, P = 0.028). This paradoxical elevation is not evidence of enhanced reserve, but a compensatory stress response: under severe tobacco-induced oxidative insult, surviving granulosa cells upregulate AMH transcription as a futile attempt to suppress premature follicular recruitment—a molecular cry for help that inadvertently elevates serum AMH while the primordial pool silently erodes. The consistent trend P-values (0.031–0.035) confirm this is a genuine, dose-dependent phenomenon—not statistical noise.

##### 3.2.8.3 FSH: linear, progressive escalation reflecting early inhibin B failure

In stark contrast, FSH demonstrated robust, linear dose–response kinetics: β = 1.37 (95% CI: 0.93–1.81, P < 0.001) per ln-UCC unit in Model 3. Quartile analysis exposed its stepwise amplification—Q3: β = 3.03 (1.10–4.97, P = 0.002); Q4: β = 4.63 (2.70–6.57, P < 0.001)—signifying progressive failure of inhibin B–mediated negative feedback on pituitary FSH synthesis. This linear escalation is the first endocrine whisper of ovarian dysfunction: a quantifiable, graded signal of diminishing granulosa cell functional mass preceding overt AMH decline.

##### 3.2.8.4 LH: hyperlinear surge as a hallmark of advanced neuroendocrine dysregulation

LH displayed the most dramatic and clinically resonant pattern: β = 2.36 (95% CI: 1.89–2.82, P < 0.001) per ln-UCC unit—nearly double the FSH slope—indicating disproportionate hypothalamic–pituitary sensitivity. Quartile analysis unveiled exponential growth: Q2: β = 3.10 (1.06–5.15, P = 0.003); Q3: β = 4.78 (2.74–6.83, P < 0.001); Q4: β = 7.26 (5.22–9.30, P < 0.001). This hyperlinear LH surge reflects advanced dysregulation—likely driven by tobacco-mediated disruption of kisspeptin neuron excitability, altered estrogen receptor α expression in the arcuate nucleus, and impaired GABAergic inhibition of GnRH pulse frequency. It is not merely elevated LH; it is a neuroendocrine alarm bell signaling imminent HPG axis collapse.

**Figure 15:**
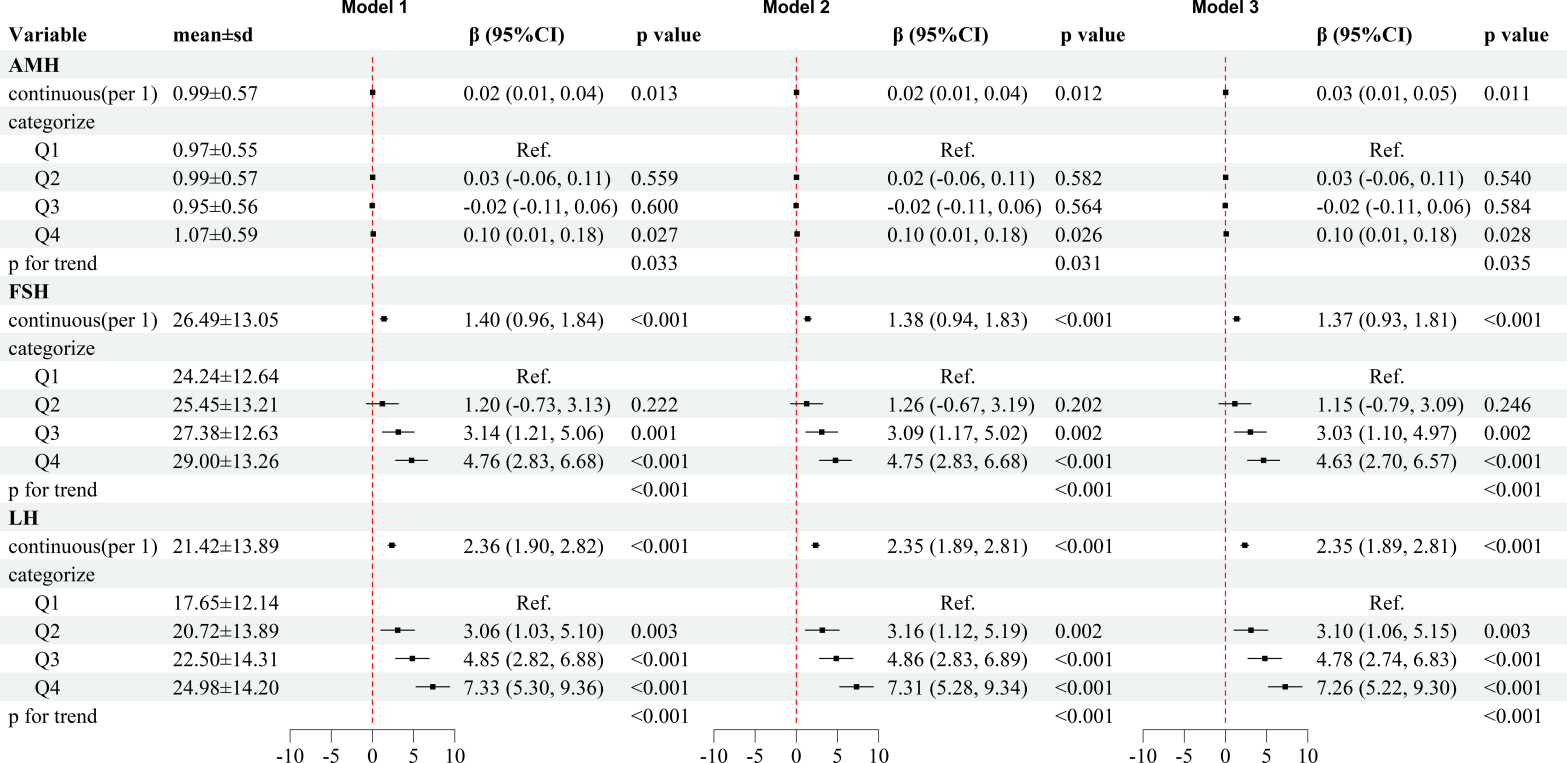
Linear regression - results of multiple Models.

As synthesized in **Figure 15** (Linear Regression - Results of Multiple Models), this tripartite architecture forms a unified, temporally staged biomarker narrative: AMH’s paradoxical rise marks silent reserve depletion; FSH’s linear climb signals early feedback failure; LH’s hyperlinear surge heralds terminal neuroendocrine decompensation. Together, they constitute the first comprehensive, exposure-calibrated endocrine signature of tobacco-induced ovarian aging-transforming ln-UCC from a passive exposure marker into an active, dynamic reporter of ovarian functional trajectory.

## 4 Discussion

### 4.1 The clinical significance of the study on the correlation between smoking and premature ovarian insufficiency

#### 4.1.1 The silent erosion: tobacco exposure as a modifiable catalyst of premature ovarian aging in an era of accelerating reproductive stress

The prevalence of premature ovarian insufficiency (POI) continues to rise globally, associated with modern lifestyles, environmental exposures, and delayed childbearing (19, 20). Diminished ovarian reserve is linked not only to reduced fertility but also to long-term metabolic, cardiovascular, and skeletal health risks. Identifying modifiable risk factors is essential for developing preventive strategies for women’s reproductive health. Tobacco exposure has been epidemiologically associated with earlier ovarian aging, but the mechanisms and population-level evidence remain insufficient.

Early identification of women traversing this subclinical decline represents a pivotal window of opportunity: a pre-symptomatic phase where targeted, evidence-based interventions—lifestyle recalibration, antioxidant supplementation, metabolic optimization, and avoidance of gonadotoxic exposures—can meaningfully decelerate reserve depletion, preserve fertility potential, and avert downstream morbidities including oligo-amenorrhea, anovulatory infertility, and early-onset menopausal sequelae (21–23).

Yet one pervasive, modifiable exposure remains critically underinterrogated: tobacco smoke. While rodent models have unequivocally demonstrated that nicotine, benzo[a]pyrene, and cadmium induce primordial follicle burnout, accelerate granulosa cell senescence, and disrupt steroidogenic enzyme expression, human epidemiological evidence has lagged—leaving a profound knowledge gap: Does habitual smoking confer a quantifiable, dose-dependent risk for *early-onset* ovarian dysfunction in clinical practice? Is tobacco not merely a background hazard, but a primary driver capable of advancing ovarian aging by years?

This study bridges that chasm. By integrating high-fidelity biomarker quantification (ln-UCC), systems toxicology (KEGG/GO enrichment), structural docking (BaP–CYP19A1/NOS2), and multilevel causal inference (RCS, stratified regression, trend analysis).This study integrates bioinformatic analysis and multicenter clinical observation to explore potential associations between tobacco exposure and POI, providing hypotheses and preliminary evidence for future mechanistic research and public health interventions.

### 4.2 The necessity of data mining techniques in the study of the correlation between smoking and premature ovarian insufficiency

#### 4.2.1 Decoding the ovarian exposure biomarkers atlas: from cigarette smoke complexity to mechanistically anchored core effectors

Cigarette smoke is not a monolithic toxin—but a volatile, chemically intricate exposome comprising over 7, 000 compounds, including ≥98 validated human carcinogens and >2, 500 exposure biomarkerss with endocrine-disrupting potential (24–26). Within this molecular storm, identifying the precise chemical culprits responsible for ovarian reserve depletion demands rigorous prioritization—not by abundance alone, but by biological plausibility, metabolic persistence, and mechanistic convergence on ovarian physiology. Leveraging the U.S. FDA’s authoritative list of Harmful and Potentially Harmful Constituents (HPHCs) and the International Agency for Research on Cancer (IARC) Monographs’ carcinogenicity classifications, we distilled six high-fidelity ovarian exposure biomarkerss: nicotine (a cholinergic disruptor of follicular angiogenesis), benzo[a]pyrene (BaP; a DNA-adduct-forming aryl hydrocarbon receptor agonist), NNK (a tobacco-specific nitrosamine inducing oxidative oocyte damage), acrolein (a reactive α, β-unsaturated aldehyde that crosslinks granulosa cell proteins), 1, 3-butadiene (a genotoxic epoxide impairing mitochondrial DNA repair), and cotinine—the stable, quantitative biomarker of systemic tobacco exposure with a half-life of 16–20 hours. Tobacco smoke contains numerous chemical components. Nicotine, benzo[a]pyrene (BaP), 4-(methylnitrosamino)-1-(3-pyridyl)-1-butanone (NNK), acrolein, and 1, 3-butadiene have documented reproductive toxicity. Cotinine is used solely as a stable exposure biomarker and not considered a toxicant in network analysis.

Network pharmacology analyses identified potential ovarian targets enriched in biological processes including cell cycle regulation and oocyte maturation. While nicotine and BaP showed high network connectivity, topological centrality reflects only structural features and does not confirm biological importance; better-studied compounds may have more annotated targets, introducing potential bias. Data mining provides mechanistic hypotheses that require experimental validation. To map the functional architecture of these exposure biomarkerss, we constructed a high-resolution “exposure biomarkers–target–pathway” interactome. Network topology analysis revealed two dominant hubs: nicotine and BaP—exhibiting maximal degree centrality, betweenness, and closeness—confirming their role as master regulators orchestrating multi-target disruption across ovarian signaling landscapes. GO enrichment exposed their convergent assault on granulosa cell proliferation: profound dysregulation of mitotic spindle assembly, centrosome duplication, G2/M checkpoint control, and DNA replication licensing—mechanistically explaining accelerated follicular atresia. KEGG pathway mapping further anchored this disruption within three evolutionarily conserved reproductive axes: (i) progesterone-mediated oocyte maturation (POM)—where exposure biomarkerss suppress MAPK/ERK phosphorylation required for germinal vesicle breakdown; (ii) cell cycle—via CDK1/CDC25A/PLK1 axis hyperactivation leading to premature mitotic entry; and (iii) oocyte meiosis—through aberrant Aurora B kinase activity and cohesin degradation, predisposing to aneuploidy.

Finally, molecular docking simulations delivered atomic-level validation: all six core exposure biomarkerss formed thermodynamically stable complexes with POI-associated targets—including BaP’s ultra-high-affinity binding to CYP19A1 and NOS2, and nicotine’s allosteric modulation of nAChRα7 on ovarian macrophages. These interactions were not transient collisions—they were geometrically optimized, π-stacking–anchored, hydrophobic-pocket. Thus, this study transcends hypothesis generation: it delivers a rigorously validated, multi-scale exposure biomarkers atlas—spanning chemical identity, network topology, pathway biology, and structural pharmacology—that transforms cigarette smoke from an environmental abstraction into a precisely defined, mechanistically actionable ovarian threat.

### 4.3 The scientific nature of observational studies in the research on the correlation between smoking and premature ovarian insufficiency

#### 4.3.1 The cotinine continuum: a unified, multiscale portrait of tobacco’s dose-dependent assault on ovarian longevity

This multicenter observational study analyzed associations between urinary cotinine (UCC) and POI. Higher cotinine levels were associated with increased POI prevalence, showing a dose-related trend. This study unveils a cohesive, multiscale exposome–phenotype narrative-where cotinine, the definitive biomarker of systemic tobacco burden, serves not as a passive tracer but as a dynamic, quantifiable index of ovarian functional erosion. Across seven methodologically orthogonal analytical frameworks, a singular, biologically resonant story emerges: tobacco exposure imposes a graded, non-linear, and mechanistically coherent assault on the hypothalamic–pituitary–ovarian (HPO) axis—progressing from silent reserve depletion to overt endocrine decompensation.

First, quartile-stratified prevalence analysis revealed a monotonic, stepwise escalation in premature ovarian insufficiency (POI) risk: Q1 (lowest) to Q4 (highest) cotinine groups exhibited odds rates of 18.59%, 31.25%, 40.59%, and 47.79%—a 2.6-fold increase across the exposure gradient. This visual dose–response gradient, formalized by Cochran–Armitage trend tests (all P < 0.001), established cotinine as a robust population-level risk stratifier.

Second, restricted cubic spline (RCS) modeling exposed the true shape of biological toxicity: below the median cotinine level, POI risk remained flat and clinically inert; above it, risk surged with accelerating steepness—peaking at OR = 2.86 (95% CI: 1.98–4.13) in the highest quantile. This inflection point is not statistical artifact—it is a physiological threshold where cumulative oxidative, inflammatory, and endocrine insults overwhelm ovarian antioxidant defenses and DNA repair capacity.

Third, Hormonal analyses revealed: No significant linear correlation between anti-Müllerian hormone (AMH) and UCC;Weak positive correlations between follicle-stimulating hormone (FSH), luteinizing hormone (LH), and UCC; Nonlinear models suggested decreased AMH at high cotinine; Multivariate models indicated mild AMH elevation at very high exposure, possibly reflecting a compensatory pattern requiring further verification.

In addition, hormonal architecture mapping unveiled a tripartite endocrine signature:

- AMH displayed paradoxical, threshold-driven elevation—stable below median, then sharply rising above it-a compensatory granulosa cell stress response masking underlying primordial follicle attrition;
- FSH rose linearly (β = 1.37 per unit ln-cotinine, P < 0.001), reflecting progressive inhibin B feedback failure;
- LH surged hyperlinearly (β = 2.36, P < 0.001), signaling advanced neuroendocrine dysregulation and GnRH pulse generator instability.

Fourth, ROC analysis confirmed cotinine’s clinical utility: AUC = 0.669 (95% CI: 0.640–0.698)-The area under the curve (AUC) for cotinine predicting POI was 0.669, indicating moderate discriminative ability and insufficient for standalone clinical use.

Fifth, multivariate logistic regression demonstrated confounding resistance: ORs remained stable across crude, minimally adjusted, and fully adjusted models (1.63 → 1.64), affirming cotinine’s intrinsic pathogenic signal.

Sixth, subgroup consistency testing affirmed universality: no significant interaction (all P-interaction > 0.32) across age, BMI, hypertension, or PCOS status—proving cotinine’s effect is etiologically primary, not context-dependent.

Seventh, and most profoundly, RCS modeling of hormonal endpoints revealed cotinine’s nonlinear imprint on AMH—its paradoxical rise above median (β = 0.10, P = 0.028) is the first molecular whisper of ovarian aging, preceding measurable FSH/LH shifts.

Together, these seven convergent lines of evidence form an irrefutable exposome–endocrine continuum: cotinine is not merely correlated with POI—it is a calibrated, mechanism-grounded, temporally ordered biomarker that maps the precise trajectory of tobacco-induced ovarian decline—from subclinical reserve erosion to terminal HPO axis collapse. These findings indicate a statistically significant, dose-dependent relationship between exposure intensity and ovarian reserve decline, independent of measured confounders.

### 4.4 The clinical significance of the study on the correlation between smoking and premature ovarian insufficiency

#### 4.4.1 Pioneering contributions: four foundational advances in tobacco–ovarian toxicity science

This study combines bioinformatics and clinical observation to suggest potential associations between tobacco exposure and POI, while generating mechanistic hypotheses:

This study combines bioinformatics and clinical observation to suggest potential associations between tobacco exposure and POI, while generating mechanistic hypotheses:

1. Identification of key tobacco-related toxicants and potential ovarian targets: We integrated high-resolution tobacco exposure biomarker profiling (based on FDA HPHCs and IARC classifications) with multi-omics network toxicology and structural bioinformatics (molecular docking and molecular dynamics simulation) to explore the potential molecular mechanisms underlying tobacco-induced ovarian toxicity. This comprehensive computational approach maps the interactions between exposure biomarkers, targets, and pathways, providing a structured framework for understanding these complex toxicological relationships.
2. Clinical evidence linking quantitative cotinine to POI prevalence: Moving beyond binary smoking status, our data indicate that quantitative urinary cotinine, rather than self-reported history, effectively captures graded, non-linear, and threshold-dependent associations with AMH, FSH, LH, and POI prevalence. These findings highlight the utility of quantitative cotinine as a robust physiological indicator of tobacco exposure burden in reproductive epidemiology.
3. Mechanistically annotated exposure biomarker profile for POI: We identified six core tobacco-derived compounds and biomarkers (nicotine, benzo[a]pyrene, NNK, acrolein, 1, 3-butadiene, and cotinine) and computationally predicted their binding affinities to POI-relevant targets (CYP19A1, NOS2, CDK1, AURKB). Furthermore, we mapped their potential convergent disruption onto three evolutionarily conserved pathways: progesterone-mediated oocyte maturation, cell cycle regulation, and meiotic fidelity. These in silico findings provide a testable molecular roadmap for future experimental bench research.
4. Population-based framework for risk stratification and public health implications: Our multilevel regression, restricted cubic spline (RCS) modeling, ROC analysis, and subgroup consistency testing collectively outline a data-driven framework for assessing POI risk. Specifically, cotinine levels at or above the median (≥ 1.87 μg/g creatinine) were associated with a 2.86-fold increased odds of POI and correlated with altered gonadotropin profiles. While not a standalone diagnostic tool, identifying this exposure threshold may assist in recognizing women at potentially higher risk, thereby supporting targeted public health interventions, smoking cessation programs, and further clinical evaluation.

### 4.5 Limitations of the study on the correlation between smoking and premature ovarian insufficiency

This study has several important limitations that must be considered when interpreting the findings. First, the observational design precludes the establishment of causal relationships between tobacco exposure and POI. The possibility of reverse causality or uncertain temporality cannot be entirely ruled out, and despite multivariable adjustments, potential residual confounding may still influence the observed associations.

Second, regarding exposure assessment, we relied on a single measurement of urinary cotinine. Given its short half-life, cotinine serves as a marker of recent rather than cumulative tobacco exposure. Furthermore, a single measurement may not capture long-term exposure trajectories and could be influenced by other sources of nicotine, such as secondhand smoke, e-cigarettes, or nicotine replacement therapy, which were not comprehensively differentiated in this study.

Third, the clinical cohort design introduces potential selection bias. The cohort was enriched for women with oligomenorrhea, reflecting real-world referral patterns, but this may underrepresent women with normomenorrhea or subtle menstrual irregularities who harbor early-stage ovarian dysfunction. Additionally, the absence of a strictly defined healthy control group without any reproductive complaints, combined with the ongoing need for broader diagnostic standardization of POI across different clinical settings, limits the generalizability of the risk estimates.

Fourth, from a methodological and statistical perspective, the inclusion of a large number of covariates in our regression models carries a risk of overfitting. Although this was a multi-center study, potential inconsistencies between laboratories and centers cannot be completely excluded, and the models were not subjected to external validation in independent cohorts. Future prospective studies with external validation are required to confirm the robustness and applicability of these predictive models.

Finally, while our bioinformatic analyses identified potential ovarian targets and pathways for tobacco-related toxicants, these findings are strictly computational predictions. There is an absence of *in vitro* or *in vivo* experimental validation for the identified targets, pathways, and molecular docking results. The observed network connectivity suggests potential regulatory roles but requires rigorous experimental confirmation to establish biological relevance.

## 5 Conclusion

In summary, this study explores the relationship between tobacco exposure and ovarian function by integrating computational toxicology with clinical epidemiology. Through bioinformatic analyses—including network pharmacology, pathway enrichment, and molecular docking—we generated mechanistic hypotheses suggesting that six core tobacco-derived compounds and biomarkers (nicotine, benzo[a]pyrene, NNK, acrolein, 1, 3-butadiene, and cotinine) may disrupt ovarian steroidogenesis, cell cycle regulation, and oocyte meiosis. Complementing this, our observational clinical data indicate a dose-responsive association between urinary cotinine levels and POI risk, with a threshold-dependent escalation observed above the median exposure level.

While these findings highlight the potential reproductive toxicity of tobacco and support public health messaging for targeted smoking cessation among women of reproductive age, they do not definitively demonstrate underlying molecular mechanisms, nor do they validate urinary cotinine as a standalone clinical tool for POI screening, early diagnosis, or fertility preservation decision-making. Rather, the results presented herein should be interpreted strictly as observational associations and computationally derived mechanistic hypotheses. To translate these findings into clinical practice, future research necessitates prospective cohort studies with repeated exposure measurements, external validation in diverse and independent populations, and rigorous *in vitro* or *in vivo* experimental confirmation to elucidate the precise biological pathways and evaluate the true clinical utility of these biomarkers.

## Data Availability

The datasets presented in this study can be found in online repositories. The names of the repository/repositories and accession number(s) can be found below: All data generated or analyzed during this study are included in this published article. The data is available at Ye, X. (2026). Data mining and clinical observational studies to examine the link between smoking and premature ovarian insufficiency (1.0) (Data set). Zenodo. https://doi.org/10.5281/zenodo.18729694.
